# Effectiveness of EGFR/HER2-targeted drugs is influenced by the downstream interaction shifts of PTPIP51 in HER2-amplified breast cancer cells

**DOI:** 10.1038/s41389-018-0075-1

**Published:** 2018-08-24

**Authors:** Eric Dietel, Alexander Brobeil, Claudia Tag, Stefan Gattenloehner, Monika Wimmer

**Affiliations:** 1 Institute of Anatomy and Cell Biology, Justus-Liebig-University, Giessen, 35392 Germany; 2 Institute of Pathology, Justus-Liebig-University, 35392 Giessen, Germany

## Abstract

Breast cancer is the most common female cancerous disease and the second most cause of cancer death in women. About 20–30% of these tumors exhibit an amplification of the HER2/ErbB2 receptor, which is coupled to a more aggressive and invasive growth of the cancer cells. Recently developed tyrosine kinase inhibitors and therapeutic antibodies targeting the HER2 receptor improved the overall survival time compared with sole radio- and chemotherapy. Upcoming resistances against the HER2-targeted therapy make a better understanding of the receptor associated downstream pathways an absolute need. In earlier studies, we showed the involvement of Protein Tyrosine Phosphatase Interacting Protein 51 (PTPIP51) in the mitogen-activated protein kinase (MAPK) pathway. The MAPK pathway is one of the most frequently overactivated pathways in HER2-amplified breast cancer cells. This study is aimed to elucidate the effects of four different TKIs on the interactome of PTPIP51, namely with the receptors EGFR and HER2, 14-3-3/Raf1 (MAPK pathway), its regulating enzymes, and the mitochondria-associated interaction partners in HER2 breast cancer cell lines (SK-BR3 and BT474) by using the Duolink proximity ligation assay, immunoblotting and knockdown of PTPIP51. Inhibition of both EGFR and HER2/ErbB2R shifted PTPIP51 into the MAPK pathway, but left the mitochondria-associated interactome of PTPIP51 unattended. Exclusively inhibiting HER2/ErbB2 by Mubritinib did not affect the interaction of PTPIP51 with the MAPK signaling. Selective inhibition of HER2 induced great alterations of mitochondria-associated interactions of PTPIP51, which ultimately led to the most-effective reduction of cell viability of SK-BR3 cells of all tested TKIs. The results clearly reveal the importance of knowing the exact mechanisms of the inhibitors affecting receptor tyrosine kinases in order to develop more efficient anti-HER2-targeted therapies.

## Introduction

The identification of targetable signal nodes and protein–protein interactions is of utmost interest for the development of novel drugs for the treatment of cancer and other diseases such as neurodegenerative diseases. The human EGFR-related receptor 2 (HER2) oncogene/oncoprotein represents a perfect example of such a treatable target. The amplification of HER2 in breast cancer leads to severe alterations in growth and proliferation signaling, e.g., mitogen-activated protein kinase (MAPK) signaling, resulting in a more aggressive and invasive growth of the tumor^1,2^. Owing to the development of small molecules and therapeutic antibodies against this target, the treatment of HER2-amplified breast cancer made great progress. The combination of anthracyclin-based and non-anthracyclin-based chemotherapies with trastuzumab, a HER2-targeted therapeutic antibody, led to disease-free survival rates at 5 years of 81–84% compared with 75% without trastuzumab in HER2-positive early-stage breast cancer^3^. The already clinically established tyrosine kinase inhibitor Lapatinib, which targets epidermal growth factor receptor (EGFR) and HER2, improved the time to progression from 4.4 months to 8.4 months in a capecetabin vs. capecetabine plus lapatinib setting^4^.

HER2, also known as ErbB2 (erythroblastosis homolog B2), is an orphan receptor. It belongs to the Her family like the EGFR. As there is no identified ligand of the HER2 receptor, the downstream signaling is activated by autophosphorylation through the formation of homodimers or heterodimers with other members of the Her family. HER2 signaling is channeled into the MAPK and PI3K/Akt signaling leading to proliferation, growth, and survival of the cell.

In consequence of its upstream position, the blockage of the growth and proliferation signaling on the HER2 level can be bypassed and the effect of the small molecule inhibitor or the therapeutic antibody, respectively, is omitted^5^. In order to develop the most-effective drugs, it is crucial to understand regulatory interactions in MAPK and PI3K/Akt signaling downstream of the receptor.

One of the MAPK pathway regulators is the protein tyrosine phosphatase interacting protein 51 (PTPIP51). PTPIP51 is expressed in many highly differentiated tissues and often deregulated in cancer. It is involved in many diverse cellular functions including cell growth, differentiation, proliferation, and apoptosis. The panel of interaction partners ranges from MAPK-associated proteins (EGFR, Raf1) over scaffolding proteins (14.3.3) to NFkB signaling proteins (RelA, IkB) and mitosis-associated proteins (CGI-99, Nuf2)^6–8^.

PTPIP51 plays an essential role in the development of several cancer types. For example, the malignancy of glioblastomas is correlated to the expression of PTPIP51^9^. In basal cell and squamous cell carcinoma, the expression pattern of PTPIP51 is altered^10^. In prostate cancer, hypomethylation of the PTPIP51 promoter region results in an increased expression of the protein^11^. Malignant blasts of acute myeloid leukemia (AML) exhibit PTPIP51 expression in contrast to healthy bone marrow cells. The interaction of PTPIP51 with the MAPK pathway in AML blasts is inhibited as a result of its highly phosphorylated Tyr176 residue^12,13^.

PTPIP51 exerts its regulating effect on the MAPK pathway on Raf1 level via the scaffolding protein 14-3-3. The recruitment of PTPIP51 into the MAPK signaling leads to an activation of the MAPK pathway^7^. A well-titrated signal is a prerequisite for an optimal cellular function. Therefore, the formation of the PTPIP51/14-3-3/Raf1 complex is tightly regulated by kinases and phosphatases^12,14,15^. One of the crucial spots for this regulation is the tyrosine residue 176 of PTPIP51. Its phosphorylation results in a break-up of the PTPIP51/14.3.3/Raf1 complex and hence an omission of the MAPK signaling stimulation^14^. The phosphorylation of the Tyr176 residue is under the control of the EGFR and other kinases, such as the. c-Src kinase. Dephosphorylation is mainly performed by PTP1B^15^.

PTPIP51 is not only regulator of MAPK signaling, but also essential part of the communication site between mitochondria and endoplasmic reticulum (ER)^16,17^. These sites are called mitochondrial-associated ER membranes (MAM) and are defined by close contacts (10–30 nm) of the organelles. One of the interactions, which stabilizes these contact sites is the interaction of PTPIP51 and VAPB^18^. The communication is essential for the regulation of calcium homeostasis, apoptosis, autophagy, and many more processes, which are crucial for cell survival and cell death^19–21^. The MAMs are also signaling hubs for mammalian target of rapamycin (mTOR) and protein kinase B(Akt) signaling^22^. Alterations of these precisely regulated contact sites immensely affect the fate of the cell.

This study analyzed the effects of four different tyrosine kinase inhibitors (TKI) on the MAPK pathway, Akt signaling, and MAM-related interactome of PTPIP51 and in addition, these effects were correlated to the viability of the TKI-treated cells. We used Gefitinib, a selective EGFR inhibitor, Mubritinib, a selective HER2 inhibitor^23^, Lapatinib, an already clinically established EGFR and HER2 inhibitor, and Neratinib, a further developed EGFR and HER2 inhibitor.

## Results

### Selective inhibition of HER2 strongly affects the mitochondrial metabolism in SK-BR3 cells

To monitor the effects of selective and simultaneous inhibition of EGFR and HER2 on mitochondrial metabolism, 3-(4,5-Dimethyl-2-thiazolyl)-2,5-diphenyl-2H-tetrazolium bromide (MTT) assays were performed. SK-BR3 and BT474 cells were treated over 24 h, 48 h, or 72 h with the indicated concentration of TKIs. All cells were incubated with the same amount of dimethyl sulfoxide (DMSO) in order to exclude potential toxic effects of DMSO. The selective inhibition of HER2 by Mubritinib, especially 10 µM, reduced the mitochondrial metabolic rate of SK-BR3 cells by ~ 40%, when treated for 24 h (Fig. 2c). The effect even increased for longer incubation times (reduction of the mitochondrial metabolic rate of ~ 60% for 10 µM Mubritinib for 48 h or 72 h). BT474 cells showed a likewise behavior, when treated with the selective HER2 inhibitor Mubritinib. Although, the reduction of mitochondrial metabolic rate was not as severe as for the SK-BR3 cells (Fig. 2d).

**Fig. 1:**
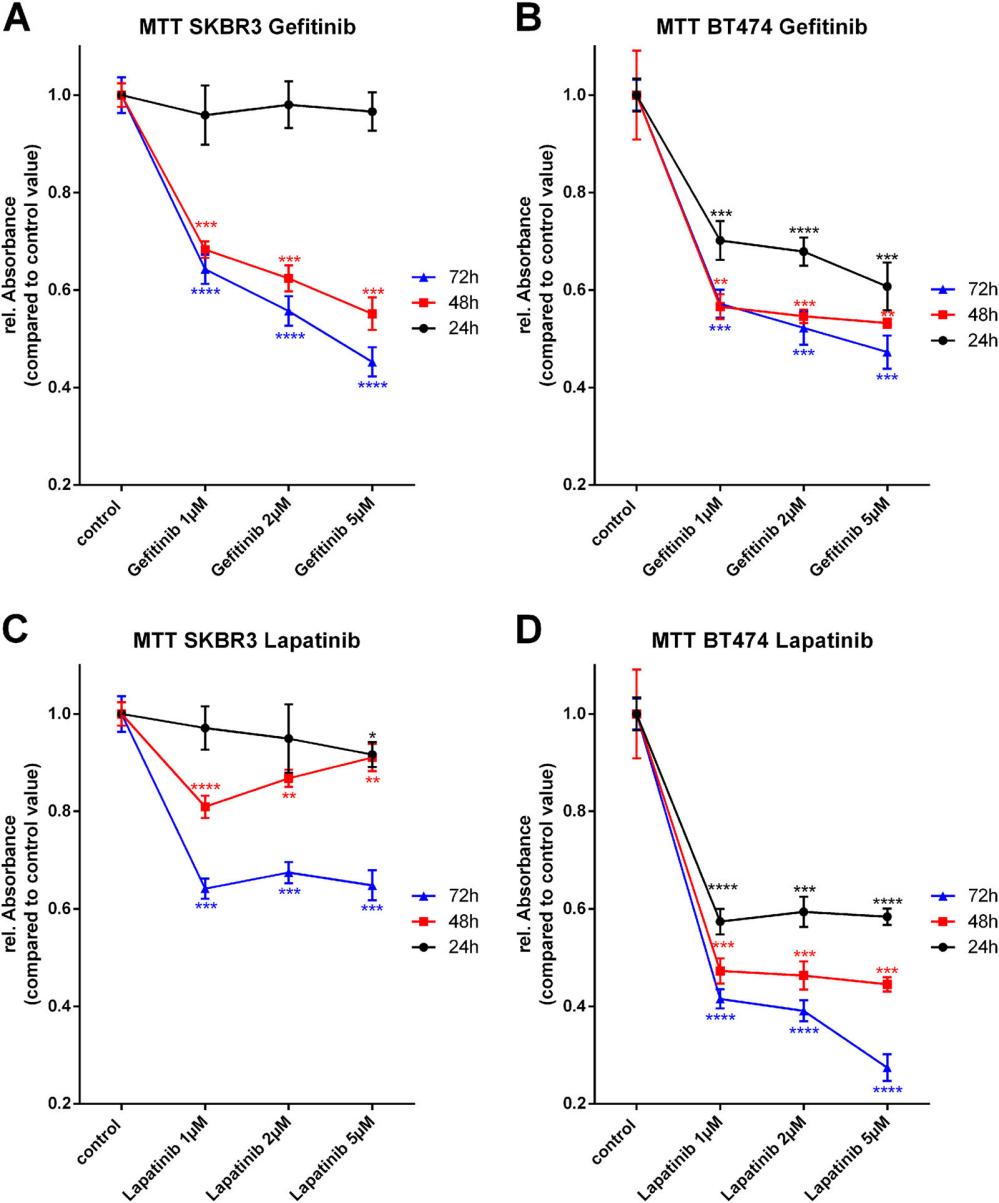
Mitochondrial metabolic rate under the influence of Gefitinib and Lapatinib. SK-BR3 cells treated with the indicated concentrations of Gefitinib over a time period of 24 h, 48 h, or 72 h (**a**). BT474 cells treated with the indicated concentrations of Gefitinib over a time period of 24 h, 48 h, or 72 h (**b**). SK-BR3 cells treated with the indicated concentrations of Lapatinib over a time period of 24 h, 48, or 72 h (**c**). BT474 cells treated with the indicated concentrations of Lapatinib over a time period of 24 h, 48 h, or 72 h (**d**). (*N* = 3)

**Fig. 2:**
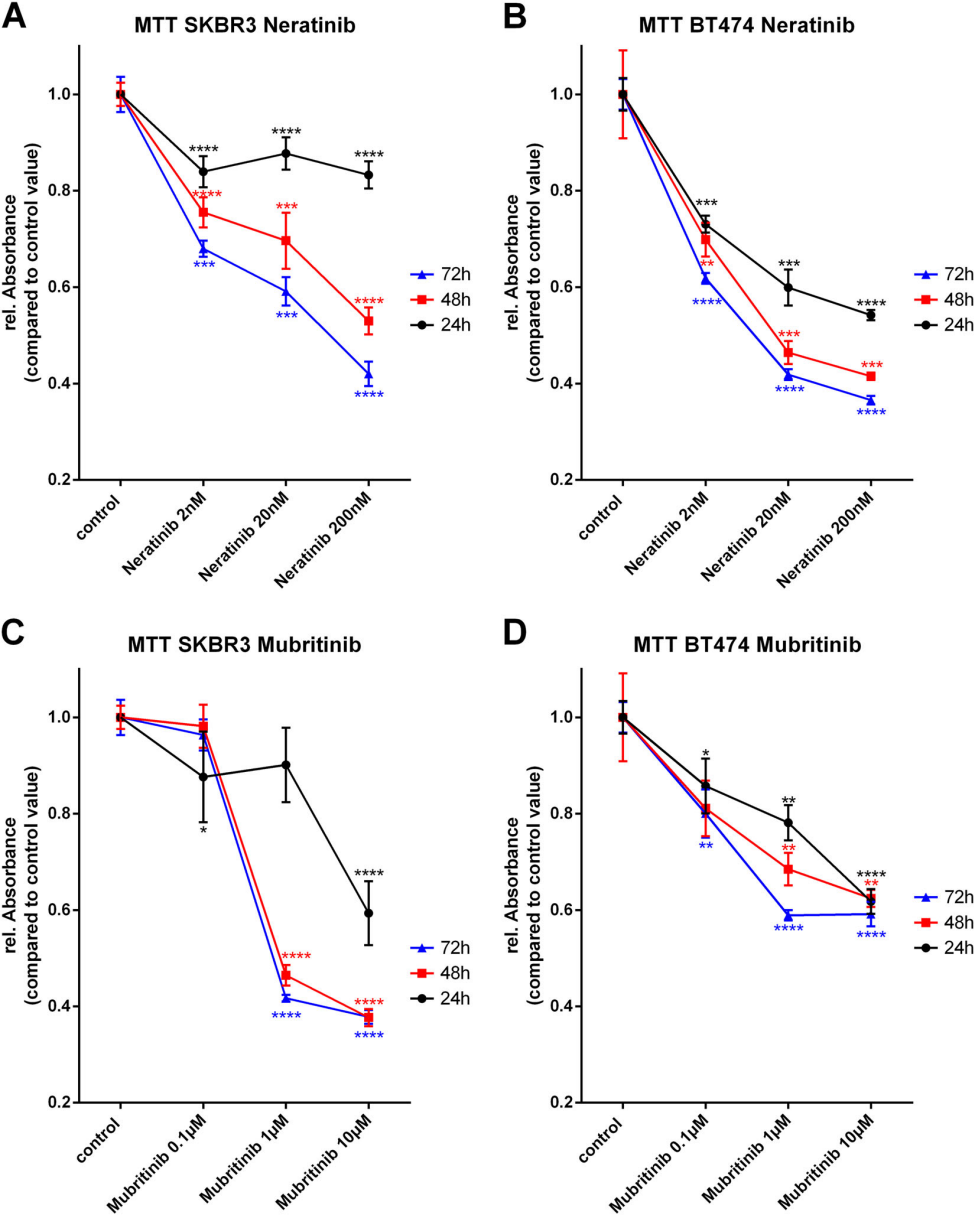
Mitochondrial metabolic rate under the influence of Neratinib and Mubritinib. SK-BR3 cells treated with the indicated concentrations of Neratinib over a time period of 24 h, 48 h, or 72 h (**a**). BT474 cells treated with the indicated concentrations of Neratinib over a time period of 24 h, 48 h, or 72 h (**b**). SK-BR3 cells treated with the indicated concentrations of Mubritinib over a time period of 24 h, 48 h or 72 h (**c**). BT474 cells treated with the indicated concentrations of Mubritinib over a time period of 24 h, 48 h, or 72 h (**d**). (*N* = 3)

The dual inhibition of EGFR and HER2 by Lapatinib and Neratinib also resulted in a significantly diminished mitochondrial metabolism but reduced the rel. Absorbance, which indicates the metabolic activity, only ~ 10–20% for SK-BR3 cells and 24 h incubation time (Figs. 1c and 2a). If incubated for 48 h or 72 h with Lapatinib or Neratinib, the reduction of the mitochondrial metabolic rate in SK-BR3 cells increased for both inhibitors. Noteworthy, application of Neratinib led to stronger reduction of mitochondrial metabolism compared with the application of Lapatinib (5 µM Lapatinib for 72 h, reduction of ~ 40%; 200 nM Neratinib for 72 h, reduction of ~ 55%). Treatment of BT474 cells with dual kinase inhibitors, Lapatinib and Neratinib, induced a reduction of mitochondrial metabolic rate of ~ 50% for the highest applied concentration and incubation time of 72 h (Fig. 1d, Fig. 2b).

The selective inhibition of EGFR by Gefitinib did not alter the mitochondrial metabolism of SK-BR3 cells if incubated for 24 h (Fig. 1a). Longer incubation times, highly significant, reduced the mitochondrial metabolism for all applied concentrations. Selective inhibition of EGFR with Gefitinib in BT474 cells induced a reduction of mitochondrial metabolism for all concentrations and incubation times (5 µM Gefitinib for 48 h or 72 h, reduction of ~ 50%) (Fig. 1b).

Noteworthy, all applied TKIs displayed a much higher reduction of metabolic activity in BT474 cells compared with SK-BR3 cells when treated for 24 h.

### Selective inhibition of EGFR leads to a formation of EGFR-HER2 dimers

For the examination of the PTPIP51 interactome under the influence of TKIs, we monitored 12 different protein–protein interactions. In order to track down the precise mode of action, we determined the formation of EGFR-HER2 dimers. Cells were treated and fixed as mentioned above. Subsequently, the cells were incubated with primary antibodies raised against EGFR and HER2, followed by Duolink proximity ligation assay (precisely described in Materials and Methods).

**Fig. 3:**
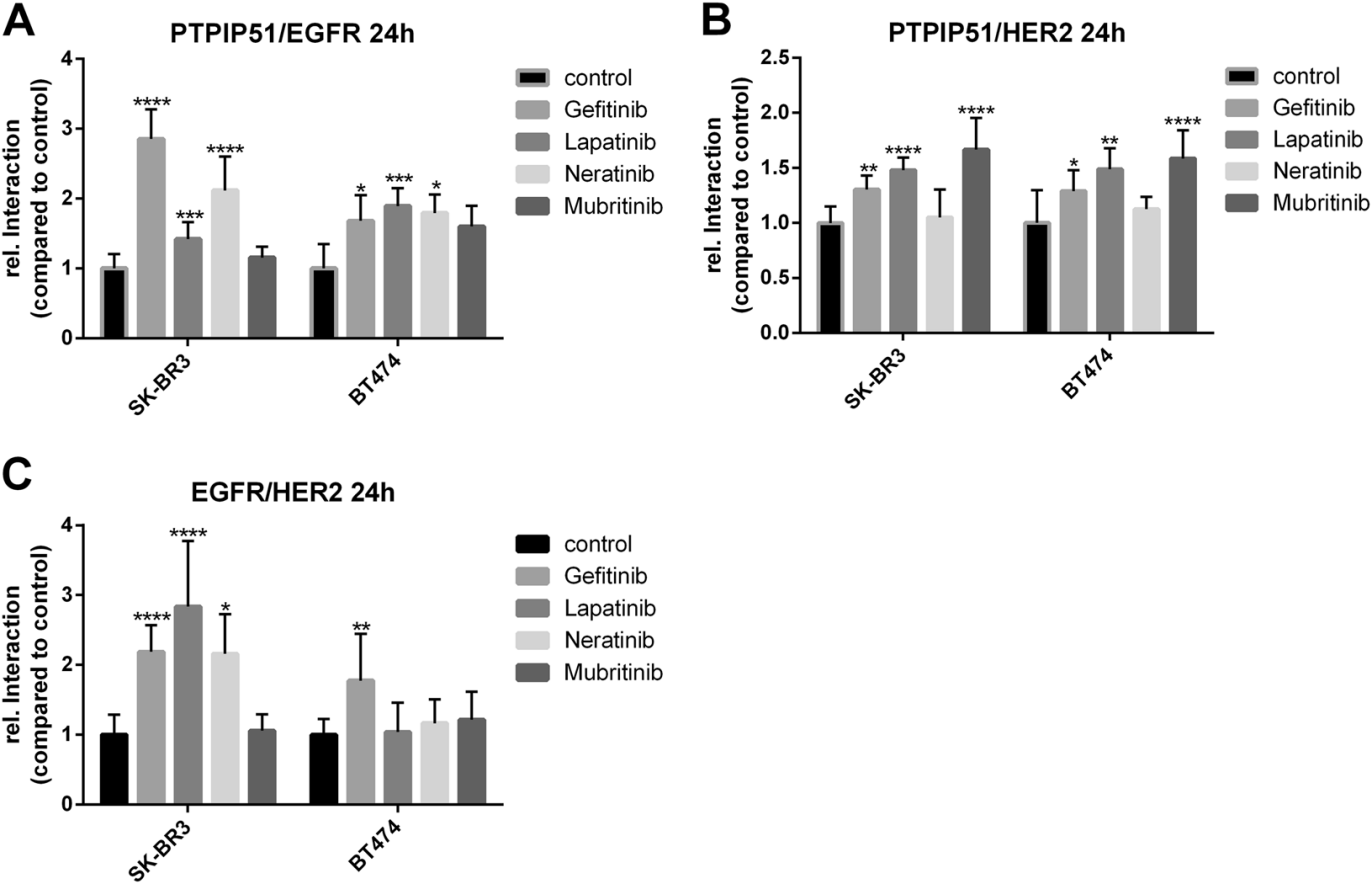
Interaction of EGFR, HER2, and PTPIP51 under Gefitinib, Lapatinib, Neratinib, and Mubritinib treatment. Interaction of EGFR and PTPIP51 in SK-BR3 and BT474 cells treated with 5 µM Gefitinib, 5 µM Lapatinib, 200 nM Neratinib, or 10 µM Mubritinib for 24 h (**a**). Interaction of HER2 and PTPIP51 in SK-BR3 and BT474 cells treated with 5 µM Gefitinib, 5 µM Lapatinib, 200 nM Neratinib, or 10 µM Mubritinib for 24 h (**b**). Interaction of EGFR and HER2 in SK-BR3 and BT474 cells treated with 5 µM Gefitinib, 5 µM Lapatinib, 200 nM Neratinib, or 10 µM Mubritinib for 24 h (**c**). (*N* = 3)

The selective inhibition of HER2 by Mubritinib did not affect the numbers of EGFR/HER2 dimers in SK-BR3 cells. Whereas the EGFR-targeted TKIs induced an enhanced formation of EGFR/HER2 dimers (Fig.3c, Supplementary Figure 1). All applied concentrations of Gefitinib induced an augmented formation of EGFR/HER2 dimers (1 µM and 2 µM *p* < 0.01, 5 µM *p* < 0.0001) (Supplementary Figure 1A). The same enhancement was observed for Lapatinib (1 µM, 2 µM, and 5 µM *p* < 0.0001) (Supplementary Figure 1B) and Neratinib (2 nM *p* < 0.0001, 20 nM *p* < 0.01, 200 nM *p* < 0.05) (Supplementary Figure 1D).

Noteworthy, in BT474 cells only the application of Gefitinib led to a significant increase of EGFR/HER2 dimers (5 µM Gefitinib *p* < 0.01) (Fig. 3c). All other applied TKI did not significantly alter the number of EGFR/HER2 dimers.

We also monitored the interactions of PTPIP51 with EGFR and HER2, respectively. The application of EGFR-targeted TKIs, Gefitinib, Lapatinib, and Neratinib, induced an enhanced interaction of PTPIP51 with EGFR and HER2. The selective inhibition of HER2 with Mubritinib only increased the interaction of PTPIP51 and HER2 (0.1 µM *p* < 0.05, 1 µM, and 10 µM *p* < 0.0001) (Supplementary Figure 2G). The interaction of EGFR and PTPIP51 remained unaffected (Supplementary Figure 2A). Likewise results were seen for BT474 cells (Fig. 3a, b)

### EGFR-targeted TKIs promote the formation of the PTPIP51–14.3.3-Raf1-complex

**Fig. 4:**
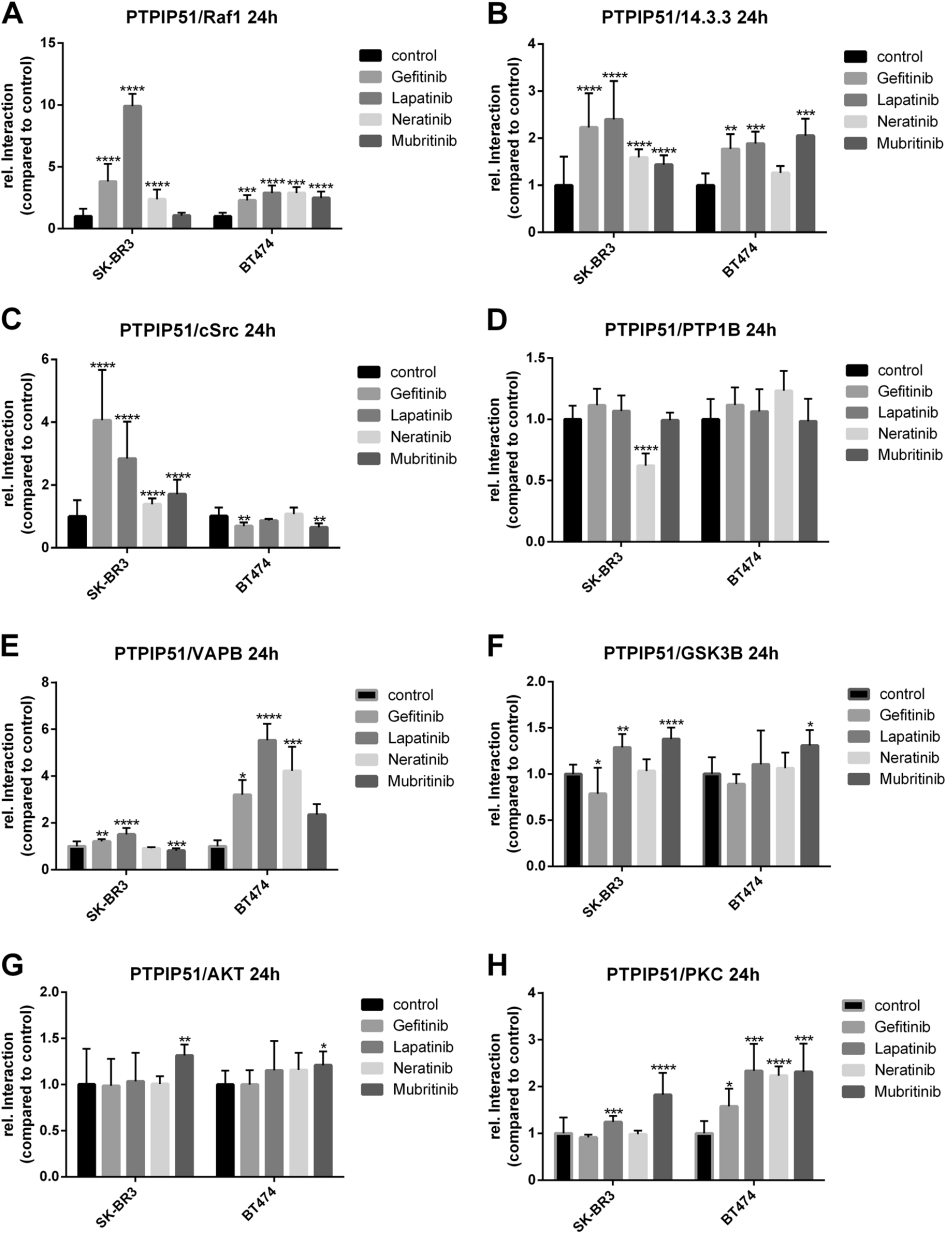
MAPK and MAM-related interactome of PTPIP51 under Gefitinib, Lapatinib, Neratinib, and Mubritinib treatment. Interaction of Raf1 and PTPIP51 in SK-BR3 and BT474 cells treated with 5 µM Gefitinib, 5 µM Lapatinib, 200 nM Neratinib, or 10 µM Mubritinib for 24 h (**a**). Interaction of 14.3.3 and PTPIP51 in SK-BR3 and BT474 cells treated with 5 µM Gefitinib, 5 µM Lapatinib, 200 nM Neratinib, or 10 µM Mubritinib for 24 h (**b**)**.** Interaction of c-Src and PTPIP51 in SK-BR3 and BT474 cells treated with 5 µM Gefitinib, 5 µM Lapatinib, 200 nM Neratinib, or 10 µM Mubritinib for 24 h (**c**). Interaction of PTP1B and PTPIP51 in SK-BR3 and BT474 cells treated with 5 µM Gefitinib, 5 µM Lapatinib, 200 nM Neratinib, or 10 µM Mubritinib for 24 h (**d**). Interaction of VAPB and PTPIP51 in SK-BR3 and BT474 cells treated with 5 µM Gefitinib, 5 µM Lapatinib, 200 nM Neratinib, or 10 µM Mubritinib for 24 h (**e**). Interaction of GSK3B and PTPIP51 in SK-BR3 and BT474 cells treated with 5 µM Gefitinib, 5 µM Lapatinib, 200 nM Neratinib, or 10 µM Mubritinib for 24 h (**f**). Interaction of Akt and PTPIP51 in SK-BR3 and BT474 cells treated with 5 µM Gefitinib, 5 µM Lapatinib, 200 nM Neratinib, or 10 µM Mubritinib for 24 h (**g**). Interaction of PKC and PTPIP51 in SK-BR3 and BT474 cells treated with 5 µM Gefitinib, 5 µM Lapatinib, 200 nM Neratinib, or 10 µM Mubritinib for 24 h (**h**). (*N* = 3)

**Fig. 5:**
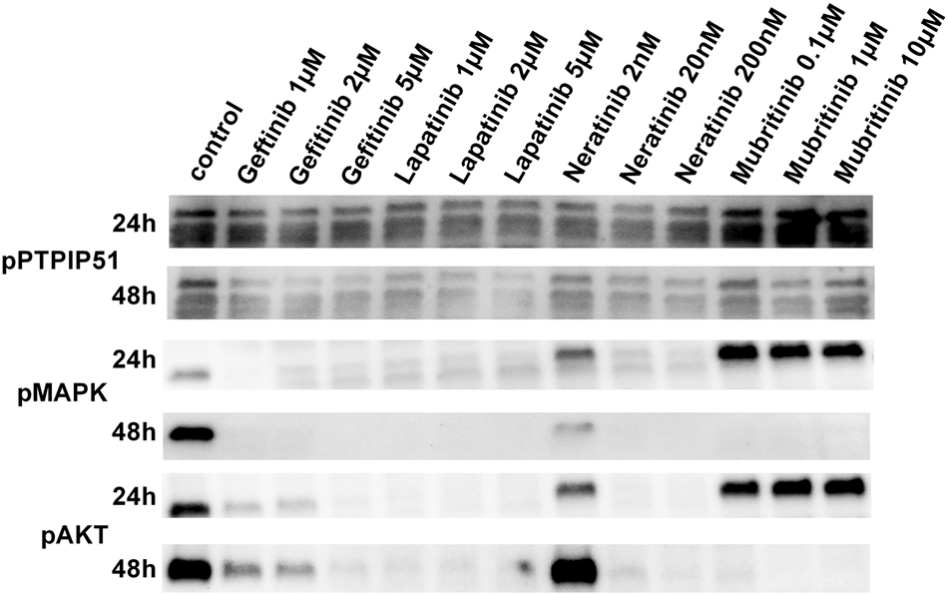
Phosphorylation status of the Tyr176 residue of PTPIP51, activation status of MAPK signaling and activation status of Akt signaling under Gefitinib, Lapatinib, Neratinib, and Mubritinib treatment. Immunobloting of phospho Tyr176 PTPIP51. Cell were treated with the indicated concentrations of the four different TKIs. The multiple bands are due to the different isoforms of PTPIP51. For evaluation only the 52 kDa isoform of PTPIP51 was used. The immunoblots were normalized to the stain-free blot

To examine the effects on MAPK-related interactions of PTPIP51 under the influence of TKI, we measured the interactions of PTPIP51 with 14.3.3 and Raf1, respectively. The inhibition of EGFR by Gefitinib, Lapatinib, or Neratinib induced the formation of the PTPIP51–14.3.3-Raf1-complex in SK-BR3 cells. Application of 2 µM and 5 µM Gefitinib applied for 24 h highly significant enhanced the interaction of PTPIP51 and 14.3.3 (*p* < 0.0001) (Fig. 4b, Supplementary Figure 3A). The complex formation of PTPIP51/Raf1 was augmented in the same manner (2 µM and 5 µM for 24 h *p* < 0.0001) (Supplementary Figure 3E). Application of Lapatinib for 24 h induced likewise observations (PTPIP51/14.3.3: 2 µM *p* < 0.001, 5 µM *p* < 0.0001; PTPIP51/Raf1 2 µM and 5 µM *p* < 0.0001) (Supplementary Figure 3B and F). Noteworthy, the complex formation of PTPIP51/14.3.3/Raf1 under Neratinib was measurable for 24 h and 48 h (PTPIP51/14.3.3: 24 h 20 nM *p* < 0.001, 200 nM *p* < 0.0001; 48 h 2 nM *p* < 0.05, 20 nM, and 200 nM *p* < 0.0001; PTPIP51/Raf1: 24 h 200 nM *p* < 0.0001; 48 h 2 nM, 20 nM, and 200 nM *p* < 0.0001) (Supplementary Figure 3D and H). On the contrary, selective inhibition of HER2 did not affect the interaction of PTPIP51 and Raf1 (Fig. 4a, b, Supplementary Figure 3H). These findings are corroborated by the observed changes of PTPIP51 Tyr176 phosphorylation. Dephosphorylation of Tyr176 of PTPIP51 is a prerequisite for the formation of the PTPIP51–14.3.3-Raf1-complex. The application of TKIs targeting EGFR resulted in a reduced phosphorylation level, whereas selective HER2 inhibition even enhanced the Tyr176 phosphorylation status (Fig. 5, Supplementary Figure 7).

The observations of formation of the PTPIP51/Raf1/14.3.3 complex under EGFR and/or HER2 inhibition in BT474 slightly differed. As seen in SK-BR3 cells, all EGFR-targeted TKI (Gefitinib, Lapatinib, and Neratinib) induced an increase of PTPIP51 and Raf1 interaction (5 µM Gefitinib *p* < 0.001, 5 µM Lapatinib, *p* < 0.0001, Neratinib *p* < 0.0001). Also Mubritinib as a selective HER2 inhibitor enhanced the interaction of PTPIP51 and Raf1 (10 µM Mubritinib *p* < 0.0001) (Fig. 4a). In addition, the interaction of PTPIP51 and 14.3.3 in BT474 cells is differently altered compared with the SK-BR3 cells. The application of Gefitinib, Lapatinib, and Mubritinib induced an increase of PTPIP51/14.3.3 interaction in BT474 (5 µM Gefitinib *p* < 0.01, 5 µM Lapatinib *p* < 0.001, 10 µM Mubritinib *p* < 0.001). In contrast to the SK-BR3 cells, Neratinib did not enhance the interaction of PTPIP51 and 14.3.3 in BT474 cells (Fig. 4b).

Besides, EGFR, which phosphorylates the Tyr176 residue, PTPIP51 phosphorylation levels are also equilibrated by the activity of the tyrosine kinase c-Src and the phosphatase PTP1B. All applied TKIs enhanced the interaction of PTPIP51 and c-Src in SK-BR3 cells (Gefitinib: 24 h 5 µM *p* < 0.0001; Lapatinib: 24 h 1 µM *p* < 0.05, 2 µM *p* < 0.01, 5 µM *p* < 0.0001, 48 h 2 µM *p* < 0.05; Neratinib: 24 h 2 nM *p* < 0.01, 20 nM *p* < 0.05, 200 nM *p* < 0.0001, 48 h 2 nM, and 200 nM *p* < 0.0001; Mubritinib: 24 h 1 µM *p* < 0.01, 10 µM *p* < 0.0001, 48 h 1 µM *p* < 0.001, 10 µM *p* < 0.0001) (Fig. 4c, Supplementary Figure 4A, B, C and D). In accordance to the differing alterations of PTPIP51/Raf1 and PTPIP51/14.3.3 interactions seen in BT474 cells, also the interaction of PTPIP51/c-Src and PTPIP51/PTP1B were affected differently. Application of Gefitinib or Mubritinib to BT474 cells reduced the interaction of PTPIP51 and c-Src (5 µM Gefitinib *p* < 0.01, 10 µM Mubritinib *p* < 0.01) (Fig. 4c). Lapatinib and Neratinib did not significantly affect the interaction of PTPIP51/c-Src in BT474 cells (Fig. 4c). The interaction of PTPIP51 with its main phosphatase PTP1B was not affected by the application of Gefitinib, Lapatinib, Neratinib, or Mubritinib in BT474 cells (Fig. 4d).

Yet, effects on the interaction of PTPIP51 with PTP1B in SK-BR3 differed. The application of Gefitinib and Lapatinib induced an increase of interaction after 48 h incubation time (Gefitinib: 1 µM *p* < 0.0001, 2 µM *p* < 0.01, 5 µM *p* < 0.001; Lapatinib: 1 µM, and 2 µM *p* < 0.0001, 5 µM *p* < 0.05) (Fig. 3d, Supplementary Figure 4E and F). On the contrary, Neratinib diminished the PTPIP51-PTP1B interaction (24 h: 20 nM *p* < 0.01, 200 nM *p* < 0.0001; 48 h: 20 nM, and 200 nM *p* < 0.001) (Supplementary Figure 4H). Mubritinib did not affect this interaction at all (Supplementary Figure 4G).

In order to determine the activation status of the MAPK signaling, pMAPK blots were performed. The inhibition of EGFR by Gefitinib, Lapatinib, or Neratinib led to a complete shutdown of MAPK signaling. Mubritinib induced an activation of MAPK signaling (Fig. 5).

### Inhibition of the EGFR and/or HER2 affects the tether between ER and mitochondria

The inhibition of EGFR led to an enhanced binding of VAPB and PTPIP51 in both cell lines(Gefitinib: 1 µM and 5 µM *p* < 0.01, 2 µM *p* < 0.001; Lapatinib: 1 µM and 5 µM *p* < 0.0001, 2 µM p < 0.05, Neratinib: 2 nM *p* < 0.0001) (Fig. 4e, Supplementary Figure 5A, B and D) in contrast to the selective inhibition of HER2, which led to a reduction of PTPIP51-VAPB interaction in SK-BR3 cells if the highest concentration of Mubritinib was applied (10 µM *p* < 0.001) (Fig. 4e, Supplementary Figure 5C). Treatment of the BT474 cells with Mubritinib did not significantly alter the number of interactions (Fig. 4e). The interaction of PTPIP51 and VAPB is regulated by the GSK3B. Lapatinib and Mubritinib induced a highly significant increase of the PTPIP51-GSK3B interaction in SK-BR3 cells (Lapatinib: 2 µM and 5 µM *p* < 0.01; Mubritinib 0.1 µM *p* < 0.05, 1 µM and 10 µM *p* < 0.0001) (Fig. 4f, Supplementary Figure 5F and G), whereas Gefitinib and Neratinib did not affect the PTPIP51-GSK3B interaction (Fig. 4f, Supplementary Figure 5E and H). In BT474 cells only the application of Mubritinib induced a significant increase of PTPIP51/GSK3B interactions (10 µM Mubritinib *p* < 0.05) (Fig. 4f).

### Inhibition of EGFR and HER2 alters the interactions of PTPIP51 with Akt and PKC

We examined the interaction of PTPIP51 with the kinase PKC and Akt. The interaction of PTPIP51 and Akt in both cell lines increased when Mubritinib was added (SK-BR3 10 µM Mubritinib *p* < 0.01; BT474 10 µM Mubritinib *p* < 0.05) (Fig. 4g). All other tested TKI did not significantly alter the interaction of PTPIP51 and Akt (Fig. 4g). The interaction of PKC and PTPIP51 significantly increased in both cell lines if Lapatinib or Mubritinib were applied (SK-BR3 5 µM Lapatinib *p* < 0.001, 10 µM Mubritinib *p* < 0.0001; BT474 5 µM Lapatinib *p* < 0.001, 10 µM Mubritinib *p* < 0.001) (Fig. 4h). Application of Gefitinib or Neratinib did not induce a significant alteration of PTPIP51/PKC interaction in SK-BR3 cell, whereas the same TKI led to an significant increase of the interaction in BT474 cells (5 µM Gefitinib *p* < 0.05, 200 nM Neratinib *p* < 0.0001) (Fig. 4h).

In order to determine the activity of Akt signaling, as an indicator of mTORC2 activity, pSer 473 Akt immunoblots were performed. The inhibition of EGFR led to an almost abolished Akt signaling. Whereas, Mubritinib induced an activation of Akt signaling as determined by its Ser473 phosphorylation (Fig. 5).

### PTPIP51 plays an essential part in the mubritinib-induced Akt activation

**Fig. 6:**
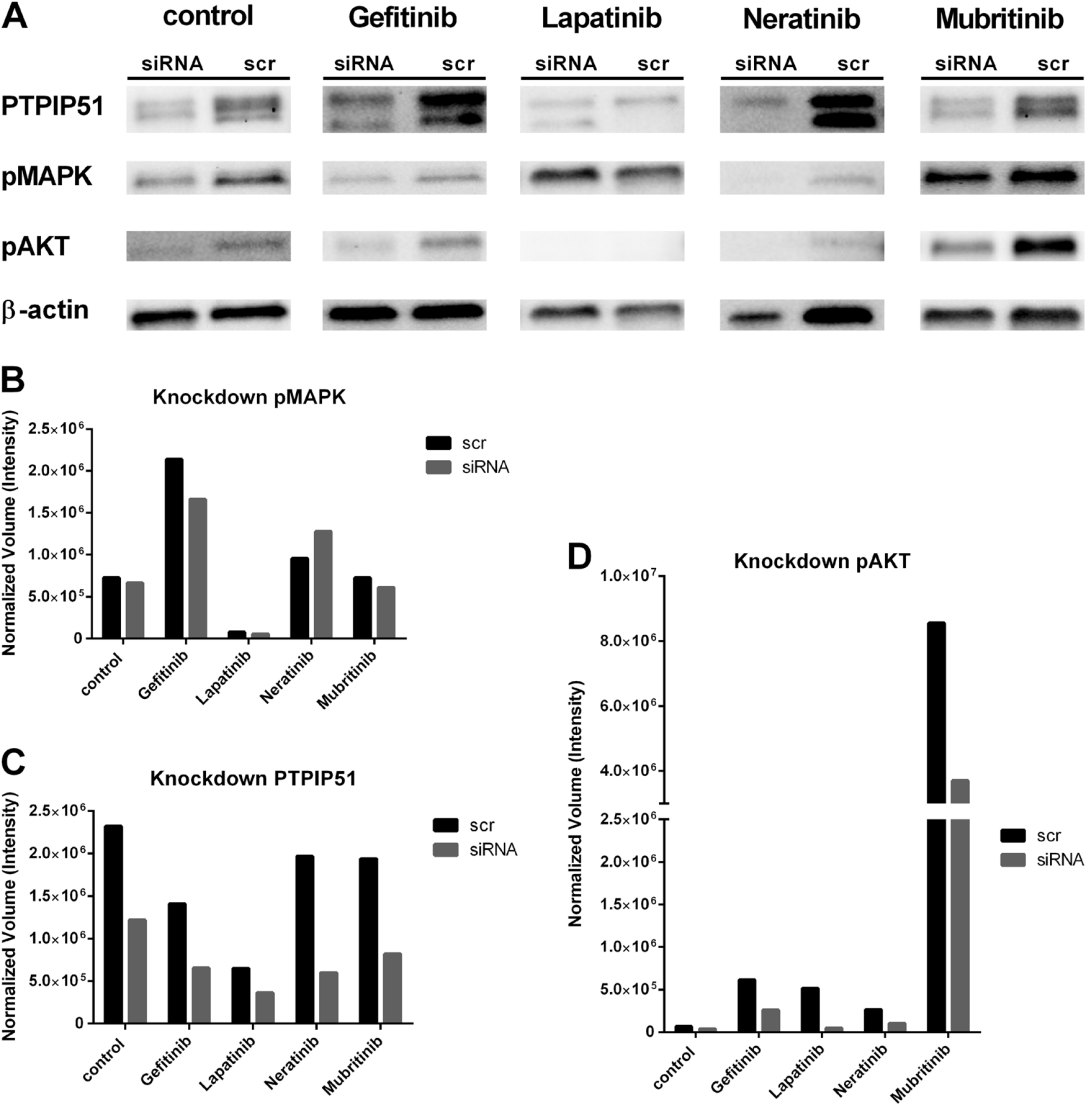
shRNA knockdown of PTPIP51 in SK-BR3 cells. Immunoblots of PTPIP51, pMAPK, and pAkt of shRNA knockdown of PTPIP51 in SK-BR3 cells treated with 5 µM Gefitinib, 5 µM Lapatinib, 200 nM Neratinib, or 10 µM Mubritinib for 24 h (**a**). Evaluation of pMAPK immunoblots (**b**). Evaluation of pAkt immunoblots (**c**). Evaluation of PTPIP51 protein immunoblots (**d**). The immunoblots were normalized to the β-Actin level

To precisely determine the importance of PTPIP51 in these complex regulations, we performed a shRNA knockdown experiment of PTPIP51 in SK-BR3 cells. The exact description of the procedure is mentioned in the Materials and Methods section. We used four different constructs for the knockdown of PTPIP51. For evaluation we only used the construct 4 since it showed the most-effective knockdown of PTPIP51 (Fig. 6a, c). The knockdown of PTPIP51 only slightly altered the activation level of the MAPK pathway as indicated by the phosphorylation of ERK1/2 (pMAPK) if no tyrosine kinase inhibitor was applied(Fig. 6a, b). Application of the tested TKI to the PTPIP51 knockdown SK-BR3 cells only slightly altered the activation of MAPK pathway (Fig. 6a, b).

Knockdown of PTPIP51 induced a strongly reduced Akt activation seen under Mubritinib treatment (Fig. 6a, d). All other tested TKI also showed a reduced Akt activation in the PTPIP51 knockdown setting (Fig. 6d).

### Selective inhibition of c-Src alters the ternary complex of PTPIP51/HER2/c-Src

**Fig. 7:**
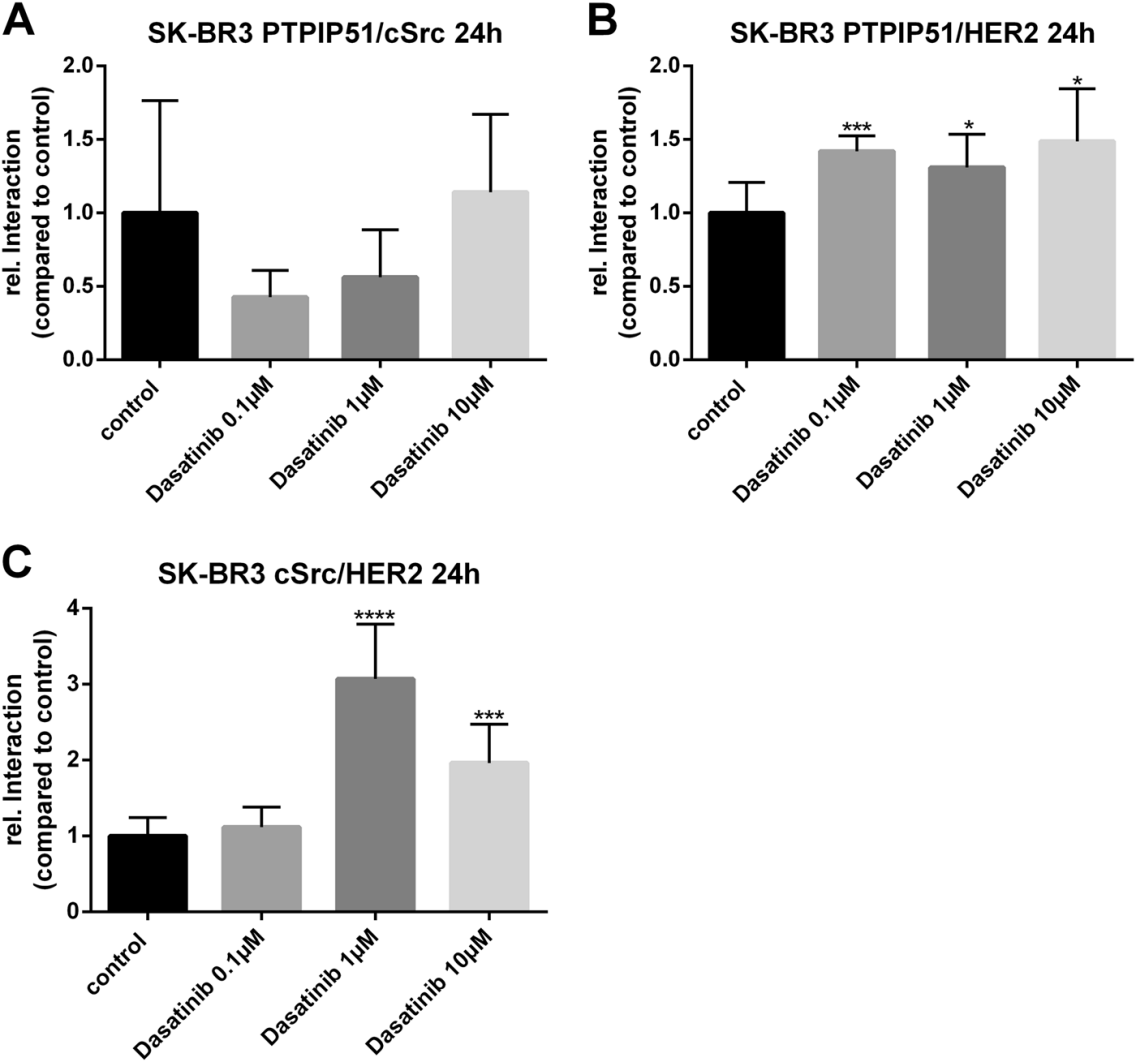
PTPIP51/HER2/c-Src interactome under selective inhibition of c-Src in SK-BR3 cells. Interaction of PTPIP51 and c-Src in SK-BR3 cells treated with the indicated concentrations of Dasatinib over 24 h (**a**). Interaction of PTPIP51 and HER2 in SK-BR3 cells treated with the indicated concentrations of Dasatinib over 24 h (**b**). Interaction of HER2 and c-Src in SK-BR3 cells treated with the indicated concentrations of Dasatinib over 24 h (**c**)

For the precise examination of the ternary complex PTPIP51/c-Src/HER2 we used Dasatinib for the selective inhibition of c-Src. Interactions were monitored using the Duolink proximity ligation assay (precisely described in Materials and Methods). Treatment of SK-BR3 cells with Dasatinib did not significantly alter the interaction of PTPIP51 and c-Src (Fig. 7a). Application of Dasatinib induced an significant increase of PTPIP51/HER2 interaction for all applied concentrations (0.1 µM *p* < 0.001, 1 µM *p* < 0.05, 10 µM *p* < 0.05) (Fig. 7b). Interaction of c-Src and HER2 was significantly enhanced if 1 µM or 10 µM Dasatinib were applied (1 µM *p* < 0.0001, 10 µM *p* < 0.001) (Fig. 7c).

## Discussion

During the last decades, targeted therapy of malignant diseases experienced a rapid development, e.g., the successful establishment of HER2-targeted therapy using the humanized monoclonal antibody Trastuzumab or TKI-like Lapatinib^24,25^. Yet, an increasing number of reports dealing with resistances to the applied targeted therapy were published^26^ The application of Trastuzumab results in a downregulation of the HER2 receptor itself, modified heterodimerization, and (de-) activation of associated signaling pathways^27^. In Trastuzumab resistant cancer cells, signaling molecules comprising PI3K and Akt as well as PTEN are dysregulated^26^. The overexpression and activation of the non-membrane tyrosine kinase c-Src was identified as a further cause for the development of resistances against HER2-targeted therapy in breast cancer cells^28^. Interestingly, the same pathways play a critical role in TKI resistance of breast cancer cells^27^.

In this context, the precise understanding of the downstream effects of EGFR and HER2 TKI is crucial to identify the affected signaling hubs and protein–protein interactions. Forming homodimers and heterodimers with EGFR and HER3, the receptor signaling of HER2 is coupled for example to the MAPK pathway, to PI3K and Akt signaling^1^. Of note, the investigated PTPIP51 represents a key player in MAPK signaling by interfering on Raf1 level^7,14,15^. The spontaneously immortalized keratinocyte cell line HaCaT displays a specific regulation and interaction pattern of PTPIP51 with the signaling molecules of the MAPK pathway^15^ If stimulated by EGF, PTPIP51 interaction with the EGFR is increased accompanied by a higher phosphorylation status of the tyrosine 176. The mechanism prevents PTPIP51 to interact with Raf1 and therefore to over-activate the MAPK pathway^15^. Moreover, PTP1B (dephosphorylates tyrosine 176 residue of PTPIP51), PKA and PKC (phosphorylate serine 212 residue of PTPIP51) interactions are augmented to maintain a basal interaction with and stimulation of Raf1 by PTPIP51^15^. The inhibition of the EGFR by Gefitinib in HaCaT cells induced an increasing interaction of PTPIP51 with 14-3-3 and Raf1 also maintaining a basal level of MAPK activity probably for cell survival^15^.

Interestingly, after 48 h incubation the inhibition of the EGFR in SK-BR3 and BT474 cells by Gefitinib, Lapatinib, and Neratinib, regardless whether or not the HER2 receptor was inhibited as well, led to a recruitment of PTPIP51 into MAPK signaling on Raf1 level particularly paralleled by the observed decrease in Tyr176 phosphorylation of PTPIP51 as seen in SK-BR3 immunoblots. In addition, the loss of Tyr176 phosphorylation was accompanied by an increased interaction of PTPIP51 and PTP1B. This interaction also facilitates the interaction of PTPIP51 with Raf1 contributing to the minimal MAPK activity of SK-BR3 cells. Therefore, we conclude that the regulatory function of PTPIP51 on the MAPK pathway is also present in both HER2-positive breast cancer cell lines SK-BR3 and BT474.

However, the tyrosine phosphorylation of PTPIP51 is still observed even though on a very low level. Inhibition of EGFR induced an enhanced interaction of PTPIP51 with the non-receptor tyrosine kinase c-Src in SK-BR3 cells, which partially compensates the inhibited EGFR kinase activity. c-Src plays a pivotal compensatory role in the loss of EGFR kinase activity in breast cancer cells and plays a crucial role in the resistance mechanism against EGFR family targeting TKI^29^. Interestingly, the application of EGFR/HER2-targeted TKI to the BT474 cells did not induce an enhancement of PTPIP51/c-Src interaction. This completely opposes the known regulations of PTPIP51, since the inhibition of EGFR is normally paralleled by an increase in PTPIP51/c-Src interaction. Furthermore, these findings correlate with a higher sensitivity of BT474 cells to EGFR-targeted TKIs as indicated by the reduction of cell viability after 24 h. There seems to be a connection between the sensitivity of HER2-positive cell lines to EGFR-targeted TKIs and the downstream regulation of the interaction of PTPIP51 and c-Src.

Besides, the potential role of PTPIP51 in the sensitivity of HER2-positive breast cancer cells to EGFR-targeted TKIs, the selective recruitment to the HER2 receptor points to a specific function of PTPIP51 in the changes of cellular signaling induced by selective HER2 inhibition. The selective recruitment is displayed by the unchanged interaction of PTPIP51 and EGFR and the unchanged heterodimer formation of the EGFR with HER2 in SK-BR3 cells. As shown for the EGFR and dual specific TKIs, Mubritinib showed an augmentation of PTPIP51/c-Src interaction in SK-BR3 cells. Of note, the interaction of PTPIP51 and c-Src gradually incremented in the same stoichiometric proportion as the PTPIP51/HER2 interaction. This depicts a potential formation of a ternary complex consisting of PTPIP51, HER2, and c-Src. This ternary interaction was influenceable by the application of the selective c-Src inhibitor Dasatinib. Interestingly, Dasatinib induced the same shift of PTPIP51 toward the HER2 receptor as seen for the application of the EGFR/HER2-targeted TKIs. Furthermore, selective c-Src inhibition also led to an increase in HER/c-Src interaction. All these findings stress an important role of PTPIP51 in the downstream regulations of the EGFR and HER2 signaling.

Beside its function in the MAPK pathway and EGFR signaling, PTPIP51 is also involved in calcium homeostasis by arbitrating the contact of the mitochondrion to the ER by the interaction with the ER bound protein VAPB^17^. The tethering interaction of PTPIP51 and VAPB is precisely regulated by the serine/threonine-kinase GSK3β. The overexpression of GSK3β leads to an impaired interaction of PTPIP51 and VAPB, whereas the inhibition enhances the interaction^18^. Of note, mitochondrial metabolism assayed by a MTT test was significantly impaired under Mubritinib treatment using 10 µM of the inhibitor paralleled by a significant reduction of the PTPIP51/VAPB interaction, indicating the decay of the MAMs in the SK-BR3 cell line. PTPIP51/GSK3β interaction was significantly enhanced under Mubritinib treatment in SK-BR3 and BT474 cells. These findings are not correlated to the observed PTPIP51/VAPB interaction data. We assume, that the PTPIP51/VAPB interaction in SK-BR3 and BT474 cells underlies various regulations and is not only determined by the activity of GSK3β. Selective inhibition of HER2 led to a significantly increased interaction of PTPIP51 with GSK3β, Akt, and PKC in SK-BR3 and BT474 cells.

The group-based prediction system (GPS 3.0; http://gps.biocuckoo.org/) identified PKC, Akt, and GSK3β to phosphorylate PTPIP51 at Ser46, located in its conserved region 1. Phosphorylation of Ser46 is the prerequisite for the PTPIP51/14.3.3. complex formation^7^. Noteworthy, the transmembrane domain of PTPIP51, which is essential for the location to the mitochondrial membrane, lies in direct vicinity to the conserved region 1. The binding of 14.3.3 at conserved region 1 probably caps the transmembrane domain of PTPIP51, thereby preventing a translocation to the mitochondrial membrane^30^. This could be a second potential regulation mechanism for PTPIP51/VAPB interaction preventing mitochondrial membrane translocation of PTPIP51 via 14.3.3 binding. The precise regulation of the PTPIP51/VAPB complex remains enigmatic and needs to be investigated in further studies.

As a functional consequence, the loss of PTPIP51/VAPB is a prerequisite for autophagy. Induction of autophagy in MCF7 breast cancer cells leads to an impaired survival, suggesting a potential tumor supportive effect of autophagy^31^. Here, the PTPIP51/VAPB interaction pattern may be predictive for a more aggressive tumor biology.

As mentioned above, the interaction of PTPIP51 and VAPB forms a physical tether between mitochondrion and ER^17^. These contact sites, namely MAM, represent important signaling hubs, e.g., for mTOR and Akt signaling. The disruption of the MAMs leads to severe alterations in the aforementioned signaling pathways and the regulation of the calcium homeostasis between mitochondrion and the ER^32,33^.

As seen here, that Akt signaling is activated under selective HER2 inhibition in SK-BR3 cells as indicated by the elevated S473 phosphorylation of Akt paralleled by a reduced interaction of PTPIP51/VAPB. This activation of Akt signaling is mediated through PTPIP51, as disclosed in the knockdown experiments. The augmented Akt phosphorylation at S473 might indicate an activation of mTORC2 signaling^34^. In addition, activation of mTORC2 signaling leads to an activation of PKC^35^. PKC in turn can activate the MAPK pathway on Raf1 level^36^. This cross-talk to MAPK signaling depicts a potential explanation of MAPK signaling activation under selective HER2 receptor inhibition. Still, the exact mechanism of how the HER2-targeted TKI Mubritinib is capable of altering the PTPIP51/VAPB interaction and the mTORC2 activity remains elusive and needs further investigations.

To sum up, PTPIP51 seems crucial for the downstream regulations of EGFR/HER2-targeted TKIs. We showed, that (1) PTPIP51/c-Src interaction is differently regulated in SK-BR3 and BT474 cells correlating with their particular sensitivity to EGFR-targeted TKIs, (2) selective inhibition of HER2 specifically recruited PTPIP51 to the HER2 receptor and to c-Src reflecting a probable mechanism for resistance, and (3) PTPIP51 is essential for the activation of Akt signaling under selective HER2 inhibition.

## Materials and methods

### Cell culture

We obtained the SK-BR3 cell line and the BT474 cell line from Cell Line Service (Eppelheim, Germany). The SK-BR3 cells were cultured in Dulbecco's MEM (Biochrom) supplemented with 10% fetal calf serum and 1% penicillin/streptomycin at 37 °C and 5% CO_2_ in a humidified chamber. BT474 cells were cultured in DMEM:Hams F12 (1:1) supplemented with L-glutamine, Insulin and FBS (Cell Lines Service, Eppelheim, Germany). The medium was renewed every 2–3 days. They were cultured until 70–80% confluence. Cell harvesting was performed with Accutase treatment for 10 min in a humidified chamber at 37 °C and 5% CO_2_. Subsequently, the cells were rinsed with sterile phosphate-buffered saline (PBS) and counted using a Neubauer counting chamber. The cells were seeded at a density of 30,000–40,000 per well in culture slides (Falcon CultureSlides, Corning Life Science, New York, USA, Cat.# 354108). Mycoplasm infection was excluded with 4′,6-diamidino-2-phenylindole (DAPI) staining for each test.

### shRNA experiments

shRNA constructs were obtained from Origene (Rockville, MD, USA). SK-BR3 and BT474 cells were grown in flat-bottomed 24-well tissue culture plates for 24 h with a starting cell number of 100,000 cells per well before transfection. The provided shRNA constructs were initially dissolved in the supplied shRNA dilution buffer. For shRNA experiments the transfection was performed using Viromer Red (Lipocalyx GmbH, Halle, Germany, Cat.# VR-01LB-01) according to the manufacturer’s protocol. The shRNA constructs were diluted to a final working dilution of 1 ng/ml.

After transfection, cells were allowed to grow for another 24 h. Transfection rate was controlled by monitoring of expression of GFP via fluorescence microscopy. Transfected cells were incubated for another 24 h with the indicated TKI. The reaction was terminated with NuPAGE LDS Sample Buffer (Thermo Fischer Scientific, Waltham, MA, USA).

### Treatment

The cells were allowed to grow for 24 h after seeding. Subsequently, they were treated with different concentrations of Gefitinib (Biaffin, Kassel, Germany, Cat.# PKI-GFTB2-200), Lapatinib (LC Laboratories, Woburn, USA, Cat.# L-4804), Neratinib (LC Laboratories, Woburn, USA, Cat.# L-6404), Mubritinib (Selleckchem, Munich, Germany, Cat.# S2216), or Dasatinib (LC Laboratories, Woburn, USA, Cat.# D-3307) for either 24 h or 48 h.

### Immunocytochemistry

The slides were washed in PBS two times for 5 min. After fixation in cold Methanol for 10 min, the slides were washed again in PBS for 8 min. The primary antibodies were diluted in blocking solution to the concentration as reported in Supplementary Table 1. After incubation at room temperature overnight under continuous movement in a humidified chamber, the slides were washed 3 × in PBS for 10 min. The secondary antibodies were diluted (Supplementary Table 1) in PBS and 10% DAPI was added. The samples were incubated for 45 min in a humidified chamber at room temperature in the dark. After washing 3 × in PBS for 10 min the slides were mounted with Mowiol and stored at 4 °C until examination.

### Duolink proximity ligation assay

To determine the interactions of proteins the Duolink Proximity ligation assay (PLA probe anti-rabbit minus, Cat.# 92005, PLA probe anti-mouse plus, Cat.# 92001; Detection Kit Orange, Cat.# 92007) was used. The assay is based on the binding of PLA probes to the primary antibodies. If these are closer than 40 nm a signal is generated. After washing the fixed SK-BR3 cells 10 min in PBS the primary antibodies diluted in blocking solution were applied (concentrations in Supplementary Table 1). The slides were allowed to incubate overnight in a humidified chamber under continuous movement. The primary antibodies were tapped off and the slides were washed in PBS 2 × 10 min. PLA probes detecting mouse (Cat# 92001–0100), goat (Cat# 92003–0100), and rabbit antibodies (Cat# 92005–0100) were diluted (1:5) in PBS. Slides were incubated at 37 °C in a humidified chamber for 1 h. The excess amount of PLA probes was tapped off and the samples were washed in Wash-Buffer A 2 × 10 min. Duolink II Ligation stock (1:5) and Duolink Ligase (1:40) were diluted in high purity water and added to the slides. After incubation for 30 min in a humidified chamber at 37 °C, the solution was tapped off and the slides were washed in Wash-Buffer A 2 × 5 min. Duolink Polymerase (1:80) and Duolink Amplification and Detection stock (1:5) were diluted in high purity water and added to the samples. The slides were allowed to incubate for 100 min in a humidified chamber at 37 °C in the dark. Finally, the slides were washed 2 × in Wash-Buffer B for 10 min and 1 × in 0.01 × Wash-Buffer B for 1 min. Nuclear staining was performed using DAPI. After drying for 30 min at room temperature in the dark they were mounted with Mowiol and stored at 4 °C until examination. Leuchowius et al.^37^ identified the Duolink proximity ligation assay as an adequate tool for identification of small molecule effectors for protein–protein interactions.

### Fluorescence microscopy

The photo documentation was performed with Axioplan 2 fluorescence microscope equipped with Plan-Apochromat objectives (Carl Zeiss Jena, Jena, Germany).

### Protein interaction analysis

For quantification, the DuoLink Image Tool (Olink Bioscience, Uppsala, Sweden, v1.0.1.2) was applied. The software identifies DAPI-positive nuclei for the cell count. Cell borders were set according to the software calculated cell shape using a user defined cell diameter preset. Fluorescence dots of the DPLA were counted by the software for each single marked cell.

### Western blot

Samples of SK-BR-3 cell lysates were separated on Mini-PROTEAN TGX Stain-Free Precast Gels (Bio-Rad, München, Germany Cat.# 4568085). The Bio-Rad Trans-Blot Turbo Transfer System (Bio-Rad, München, Germany) with the settings for mixed molecular weight proteins was used for transfer to an Immobilon-P membrane (Millipore, Billerica, USA, Cat.#IPVH07850) according to manufacturer’s instructions. The membrane was blocked with 1 × Rotiblock for 1 h h at room temperature. Incubation with anti-pMAPK or anti-Akt was done overnight at 4 °C. Horseradish peroxidase-conjugated anti-rabbit immunoglobulins diluted in 1 × Rotiblock were applied for 1 h at room temperature. The reaction was visualized with the ECL prime substrate. The Bio-Rad ChemiDoc Touch Imaging System (Bio-Rad, München, Germany) was used for documentation. Calibration was performed with a molecular weight marker suitable for chemiluminescence (Life Technologies GmbH, Darmstadt, Germany, Cat.# LC5602). The blots were equalized to the obtained stain-free blot for comparison using the Bio-Rad Image Lab (Bio-Rad, München, Germany). Hence, no loading control is required.

### MTT assay

The cells were seeded in a 96-well plate at a density of 10,000 cells per well and were allowed to grow for 24 h. The treatment of the cells was carried out as indicated. The MTT solution was added 4 h before the end of the incubation time. After formation of the formazan crystals, the solubilization solution (10% sodium dodecyl sulphate in 0.01 M HCl) was added. The solution was carried out overnight in a 37 °C 5% CO_2_ humidified chamber. Evaluation of the assay was performed with Berthold Tech TriStar ELISA Reader (Bad Wildbad, Germany).

### Statistical analysis

The data were evaluated using GraphPad Prism 6 software. For variance analysis One way analysis of variance tests were performed. Statistical testing was done using the Dunnett’s multiple comparison test in case of Gaussian distribution. Otherwise, Dunn’s multiple comparison was used. Results were considered significant with *p* < 0.05. (*(*p* < 0.05), **(*p* < 0.01), ***(*p* < 0.001), ****(*p* < 0.0001))

### Availability of data and materials

All data generated or analyzed during this study are included in this published article (and its supplementary information files).

## Electronic supplementary material

Supplemental Material
