# Supplementary material for: Effectiveness of EGFR/HER2-targeted drugs is influenced by the downstream interaction shifts of PTPIP51 in HER2-amplified breast cancer cells
Source: Oncogenesis. 2018 Aug 24;7(8):64. doi: 10.1038/s41389-018-0075-1 (PMC6107558; doi:10.1038/s41389-018-0075-1)
Supplement: Supplementary file 1 — Supplemental Material [file 41389_2018_75_MOESM1_ESM.pdf]

Table1. List of antibodies

	Immunogen	Antibody source	Clone	Dilution	Manufactor
PTPIP51(P51ab)	Human recombinant PTPIP51 protein encoding amino acids (aa) 131-470	Rabbit polyclonal		1:500	Prof. HW Hofer, Biochemical Department, University Konstanz, Germany
tyrosine 176 phosphorylated PTPIP51 (PP51)	Purified total IgG fractionKLH-peptide conjugate	Guinea pig polyclonal		1:400	BioLux, Stuttgart, Germany
Raf-1	Mapping the C-terminus of human origin	Mouse monoclonal	E-10	1:100	Santa Cruz Biotechnology Cat.# sc-7267
14-3-3β (14.3.3)	Specific for an epitope mapping between aa 220-244 at the C-terminus of 14-3-3β of human origin	Mouse monoclonal	A-6	1:100	Santa Cruz Biotechnology Cat.# sc-25276
PTP1B	epitope mapping at the N-terminus of PTP1B of human origin	Goat polyclonal	N-19	1:100	Santa Cruz Biotechnology Cat.# sc-1718
c-Src	specific for an epitope mapping between amino acids 1-30 at the N-terminus of c-Src p60 of human origin	Mouse monoclonal	H-12	1:100	Santa Cruz Biotechnology Cat.# sc-5266
GSK-3β	raised against amino acids 345-420 mapping at the C-terminus of GSK-3β of human origin	Mouse monoclonal	E-11	1:100	Santa Cruz Biotechnology Cat.# sc-377213
VAPB	E.coli-derived recombinant human VAP-B Ala2-Pro132	Mouse monoclonal	736904	1:100	R&D systems Cat.# MAB58551
Her2	ERBB2 (NP_004439, 22aa ~ 121aa) partial recombinant protein with GST tag. MW of the GST tag alone is 26 KDa	Mouse monoclonal	22-121	1:100	Abnova, Taipei, Taiwan Cat.# H0000 2064-M05
Phospho-Akt (Ser473)	a synthetic phosphopeptide corresponding to residues surrounding Ser473 of mouse Akt	Rabbit monoclonal		1:2500	Cell signaling technology #9271
Phospho-p42/p44 MAPK	a synthetic phosphopeptide corresponding to residues surrounding Thr202/Tyr204 of human p44 MAP kinase	Rabbit monoclonal		1:2500	Cell signaling technology #9111
EGFR	raised against plasma membranes of A431 cells	Mouse monoclonal	2E9	1:100	Santa Cruz Biotechnology Cat.# sc-57091
Akt	E. coli-derived recombinant human Akt1 Ser2-Ala480	Mouse monoclonal		1:100	R&D systems Cat.# MAB2055
PKC	recognizes an epitope located within the amino acid sequence 296-317, at the hinge region, close to or at the trypsin cleavage site of protein kinase C (PKC)	Mouse monoclonal	MC5	1:100	Sigma Aldrich Cat.# P5704

Supplementary Table 1 **List of antibodies used for this study.**

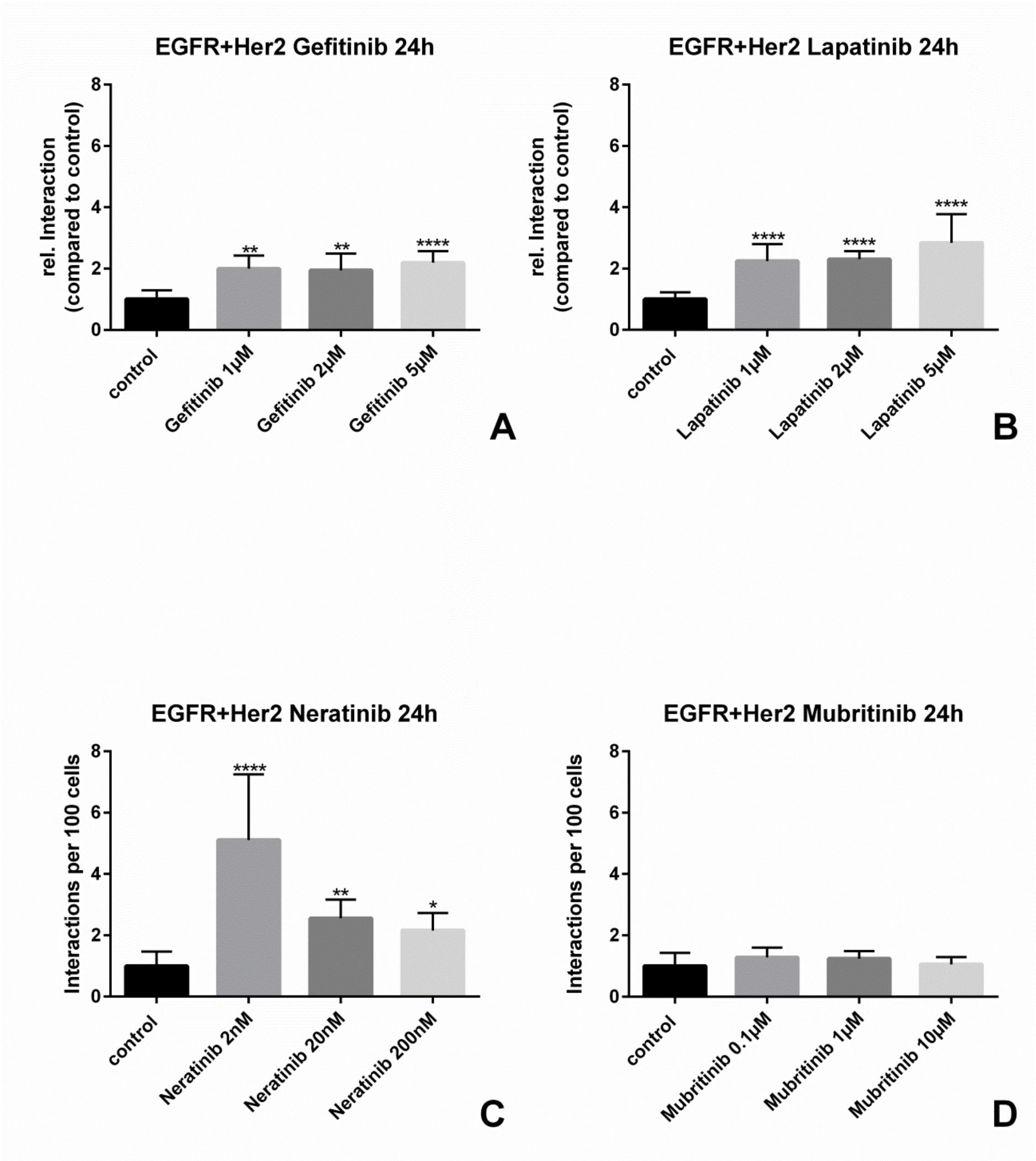

Supplementary Figure 1 **Formation of EGFR/HER2 dimers under TKI treatment.** SKBR3 cells were treated with the indicated concentrations of the 4 different tyrosine kinase inhibitors for 24h. The protein-protein interactions were measured using the Duolink proximity ligation assay. (A) Interaction of EGFR and HER2 under the influence of Gefitinib; (B) Interaction of EGFR and HER2 under the influence of Lapatinib; (C) Interaction of EGFR and HER2 under the influence of Neratinib; (D) Interaction of EGFR and HER2 under the influence of Mubritinib. (N=3)

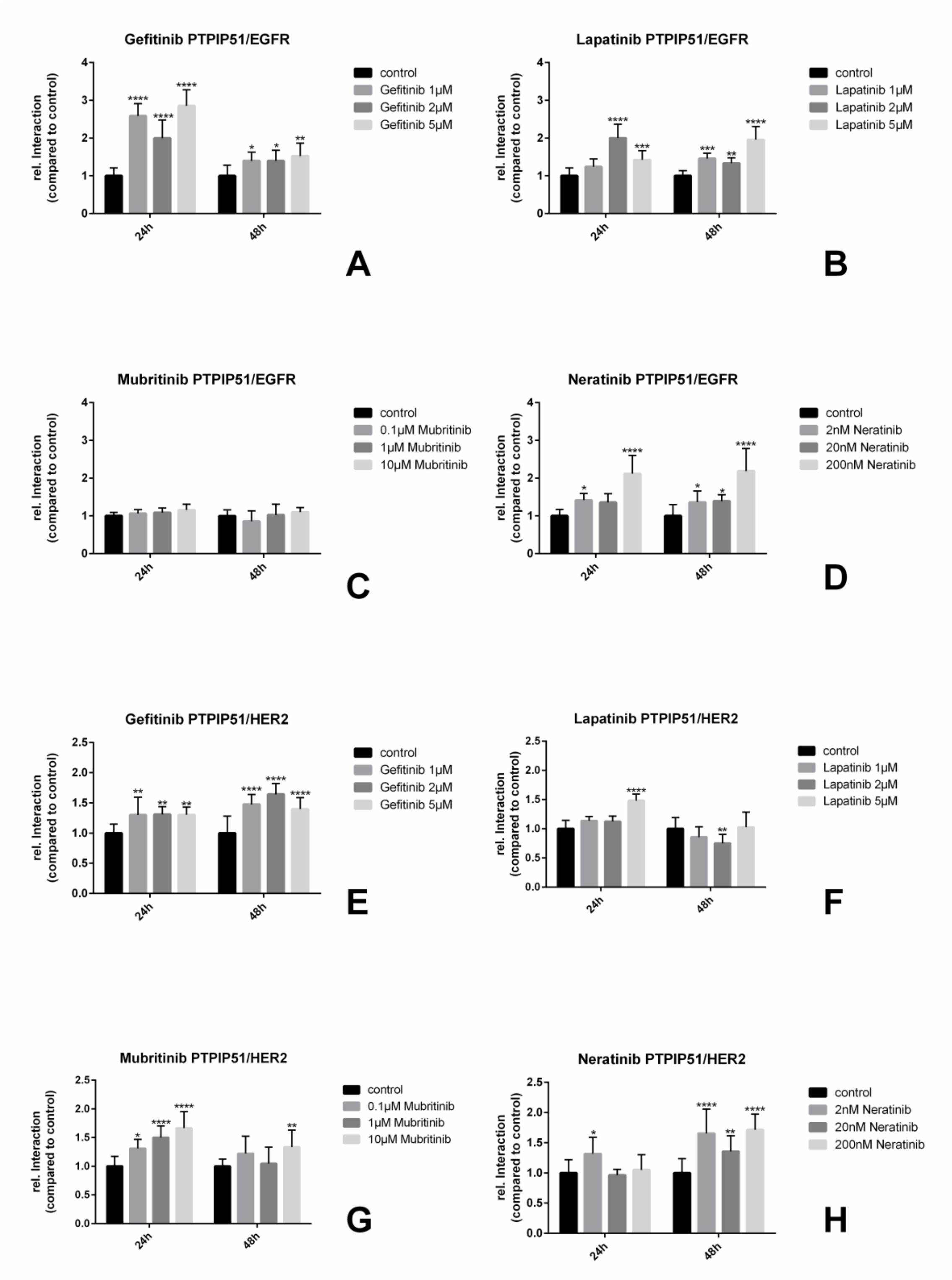

Supplementary Figure 2 **Interaction of PTPIP51 with EGFR and HER2.** SKBR3 cells were treated with the indicated concentrations of the 4 different tyrosine kinase inhibitors for 24h and 48h. The protein-protein interactions were measured using the Duolink proximity ligation assay. (A) Interaction of PTPIP51 and EGFR under the influence of Gefitinib; (B) Interaction of PTPIP51 and EGFR under the influence of Lapatinib; (C) Interaction of PTPIP51 and EGFR under the influence of Mubritinib; (D) Interaction of PTPIP51 and EGFR under the influence of Neratinib. (E) Interaction of PTPIP51 and HER2 under the influence of Gefitinib; (F) Interaction of PTPIP51 and HER2 under the

influence of Lapatinib; (G) Interaction of PTPIP51 and HER2 under the influence of Mubritinib; (H) Interaction of PTPIP51 and HER2 under the influence of Neratinib. (N=3)

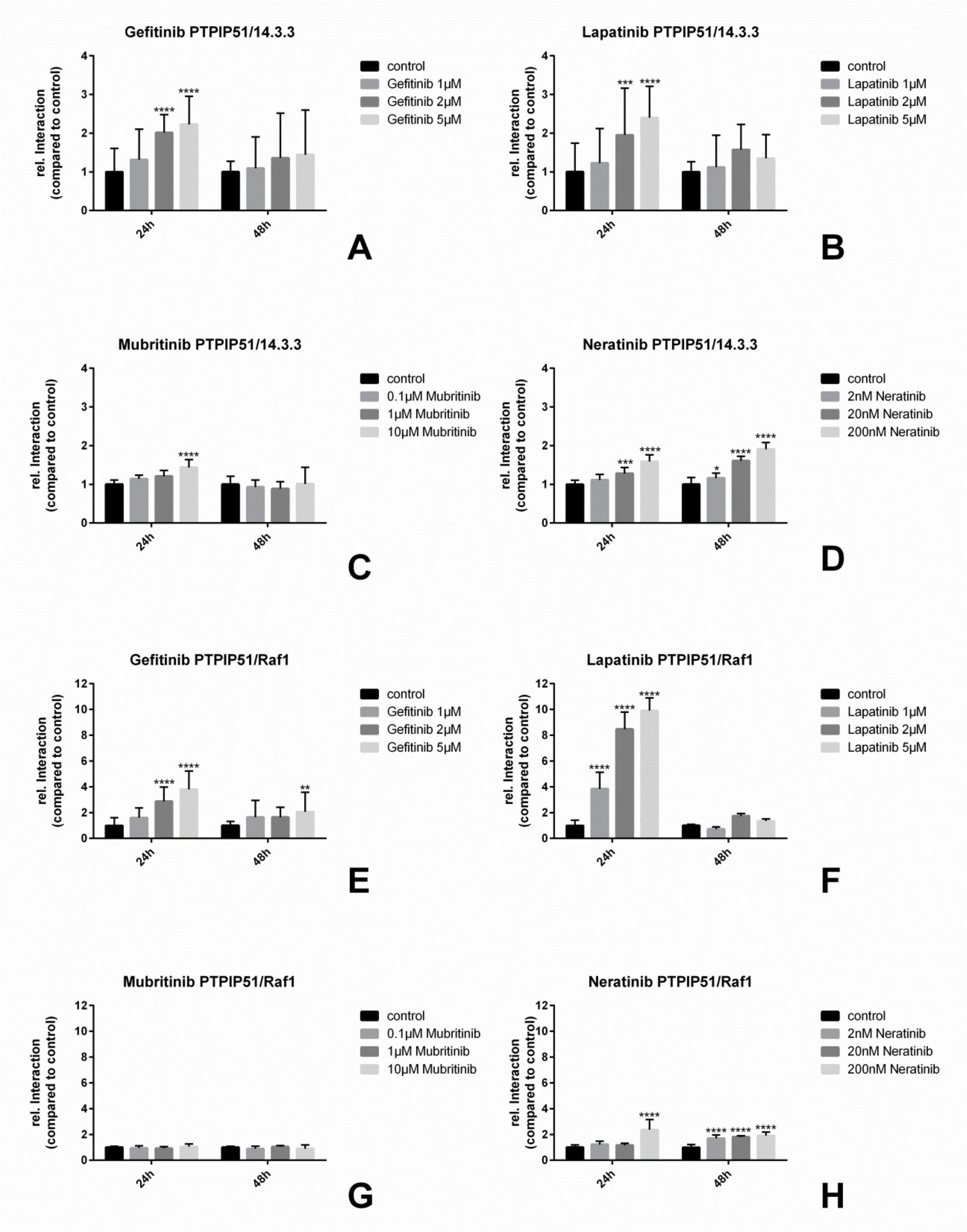

Supplementary Figure 3 **Interaction of PTPIP51 with 14.3.3 and Raf1.** SKBR3 cells were treated with the indicated concentrations of the 4 different tyrosine kinase inhibitors for 24h and 48h. The protein-protein interactions were measured using the Duolink proximity ligation assay. (A) Interaction of PTPIP51 and 14.3.3 under the influence of Gefitinib; (B) Interaction of PTPIP51 and 14.3.3 under the influence of Lapatinib; (C) Interaction of PTPIP51 and 14.3.3 under the influence of Mubritinib; (D) Interaction of PTPIP51 and 14.3.3 under the influence of Neratinib. (E) Interaction of

PTPIP51 and Raf1 under the influence of Gefitinib; (F) Interaction of PTPIP51 and Raf1 under the influence of Lapatinib; (G) Interaction of PTPIP51 and Raf1 under the influence of Mubritinib; (H) Interaction of PTPIP51 and Raf1 under the influence of Neratinib. (N=3)

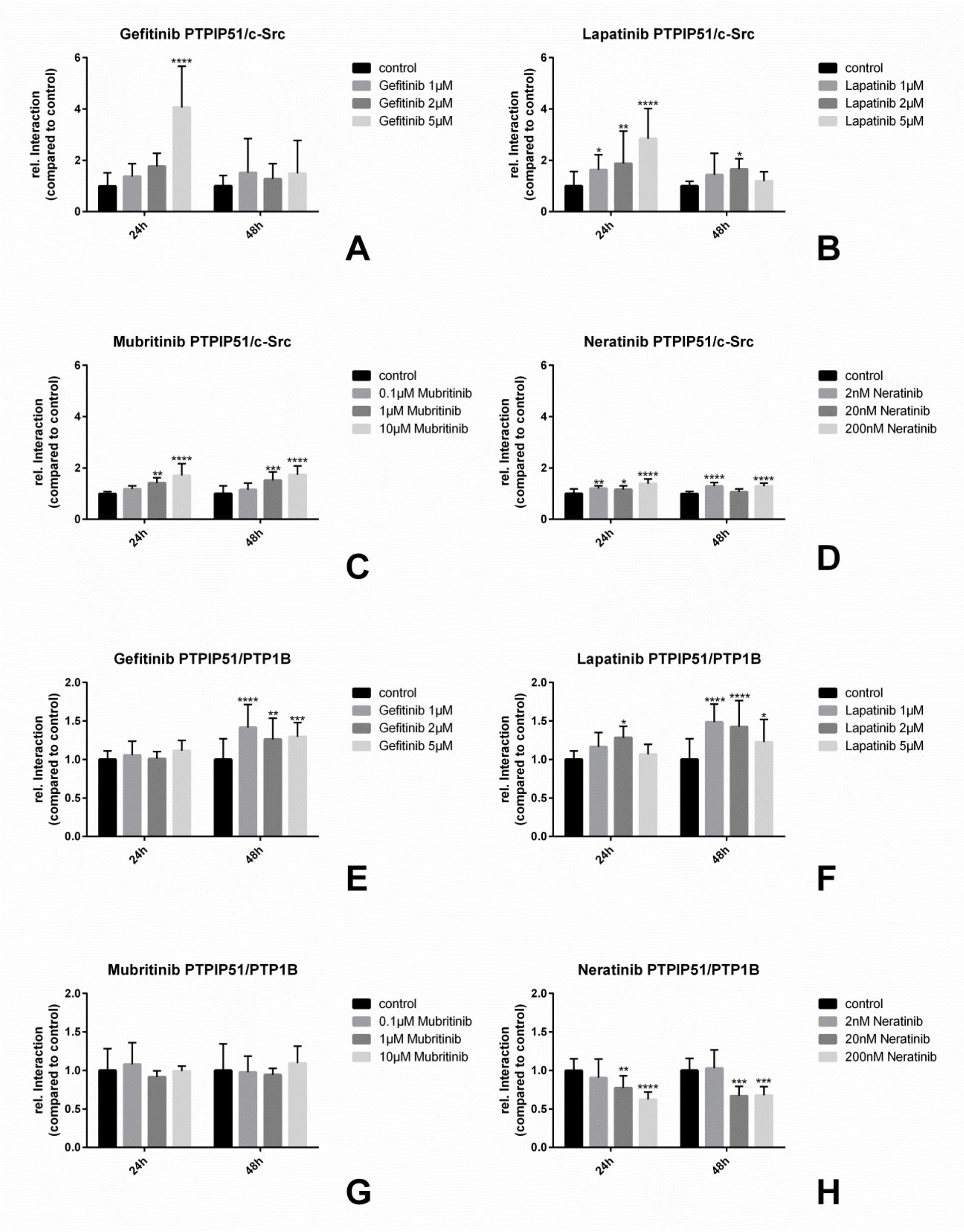

Supplementary Figure 4 **Interaction of PTPIP51 with c-Src and PTP1B.** SKBR3 cells were treated with the indicated concentrations of the 4 different tyrosine kinase inhibitors for 24h and 48h. The protein-protein interactions were measured using the Duolink proximity ligation assay. (A) Interaction of PTPIP51 and cSrc under the influence of Gefitinib; (B) Interaction of PTPIP51 and cSrc under the influence of Lapatinib; (C) Interaction of PTPIP51 and cSrc under the influence of

Mubritinib; (D) Interaction of PTPIP51 and cSrc under the influence of Neratinib. (E) Interaction of PTPIP51 and PTP1B under the influence of Gefitinib; (F) Interaction of PTPIP51 and PTP1B under the influence of Lapatinib; (G) Interaction of PTPIP51 and PTP1B under the influence of Mubritinib; (H) Interaction of PTPIP51 and PTP1B under the influence of Neratinib. (N=3)

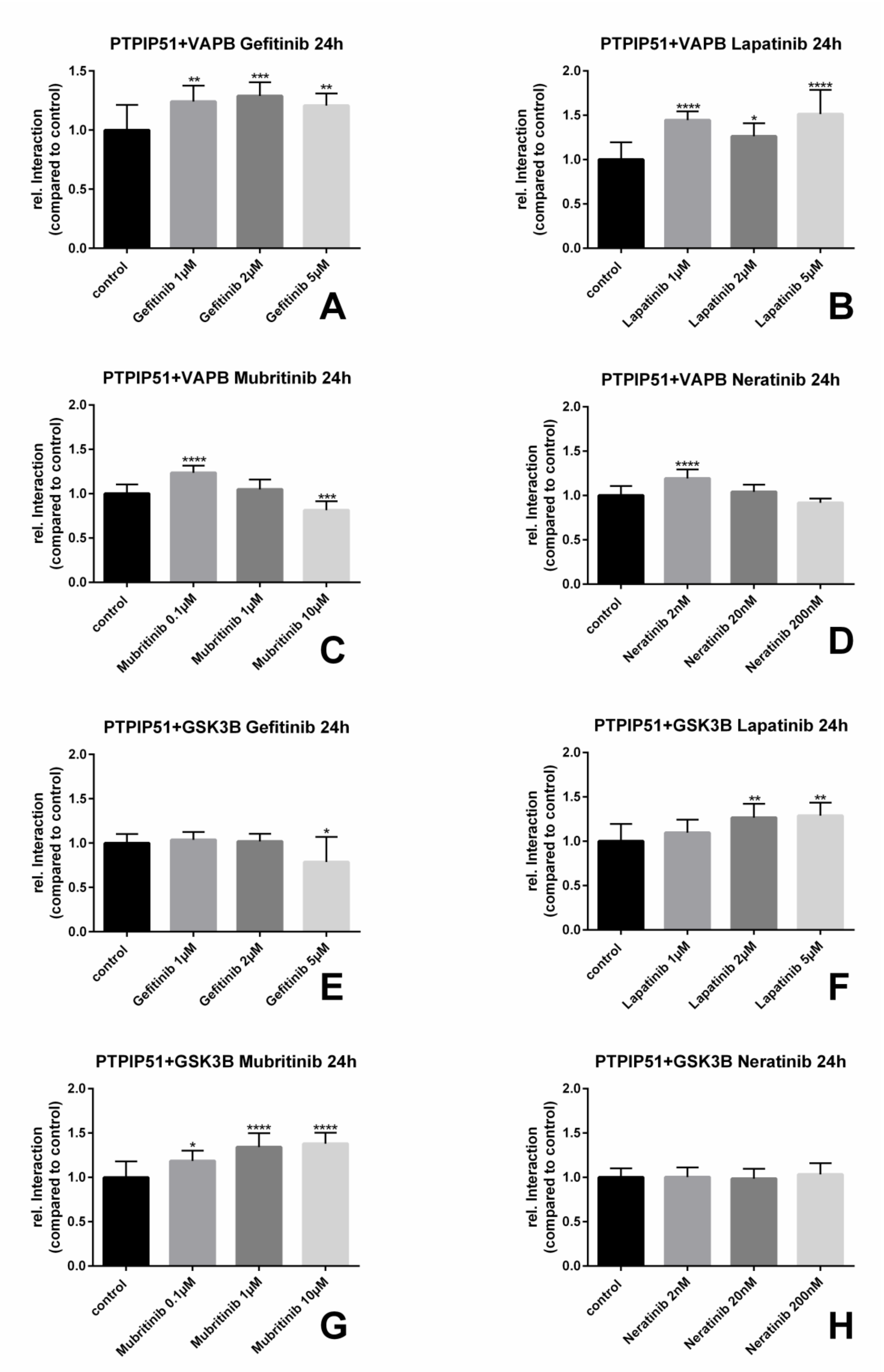

Supplementary Figure 5 **Interaction of PTPIP51 with VAPB and GSK3β.** SKBR3 cells were treated with the indicated concentrations of the 4 different tyrosine kinase inhibitors for 24h. The protein-protein

interactions were measured using the Duolink proximity ligation assay. (A) Interaction of PTPIP51 and VAPB under the influence of Gefitinib; (B) Interaction of PTPIP51 and VAPB under the influence of Lapatinib; (C) Interaction of PTPIP51 and VAPB under the influence of Mubritinib; (D) Interaction of PTPIP51 and VAPB under the influence of Neratinib. (E) Interaction of PTPIP51 and GSK3β under the influence of Gefitinib; (F) Interaction of PTPIP51 and GSK3β under the influence of Lapatinib; (G) Interaction of PTPIP51 and GSK3β under the influence of Mubritinib; (H) Interaction of PTPIP51 and GSK3β under the influence of Neratinib. (N=3)

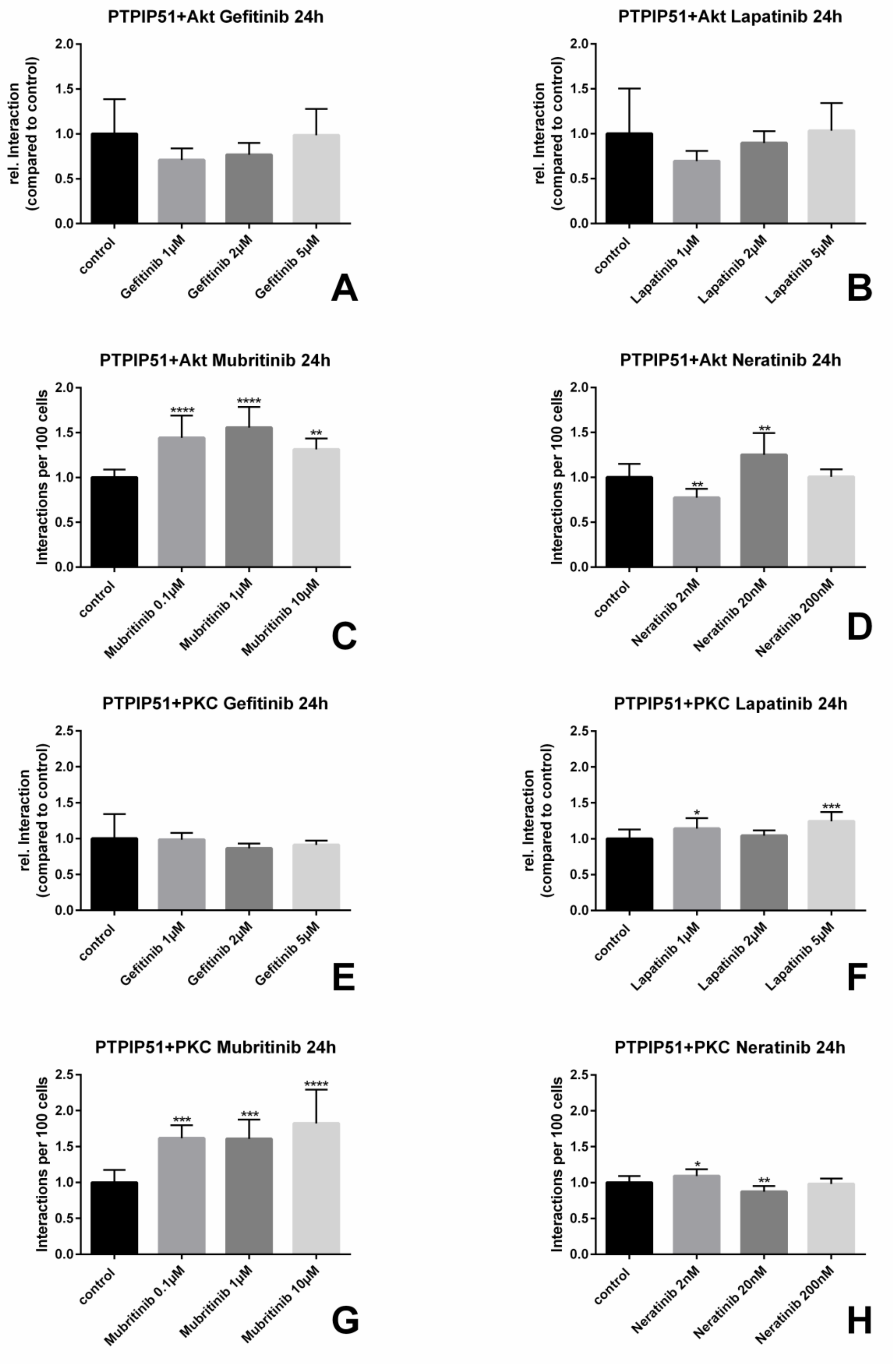

Supplementary Figure 6 **Interaction of PTPIP51 with Akt and PKC.** SKBR3 cells were treated with the indicated concentrations of the 4 different tyrosine kinase inhibitors for 24h. The protein-protein

interactions were measured using the Duolink proximity ligation assay. (A) Interaction of PTPIP51 and Akt under the influence of Gefitinib; (B) Interaction of PTPIP51 and Akt under the influence of Lapatinib; (C) Interaction of PTPIP51 and Akt under the influence of Mubritinib; (D) Interaction of PTPIP51 and Akt under the influence of Neratinib. (E) Interaction of PTPIP51 and PKC under the influence of Gefitinib; (F) Interaction of PTPIP51 and PKC under the influence of Lapatinib; (G) Interaction of PTPIP51 and PKC under the influence of Mubritinib; (H) Interaction of PTPIP51 and PKC under the influence of Neratinib. (N=3)

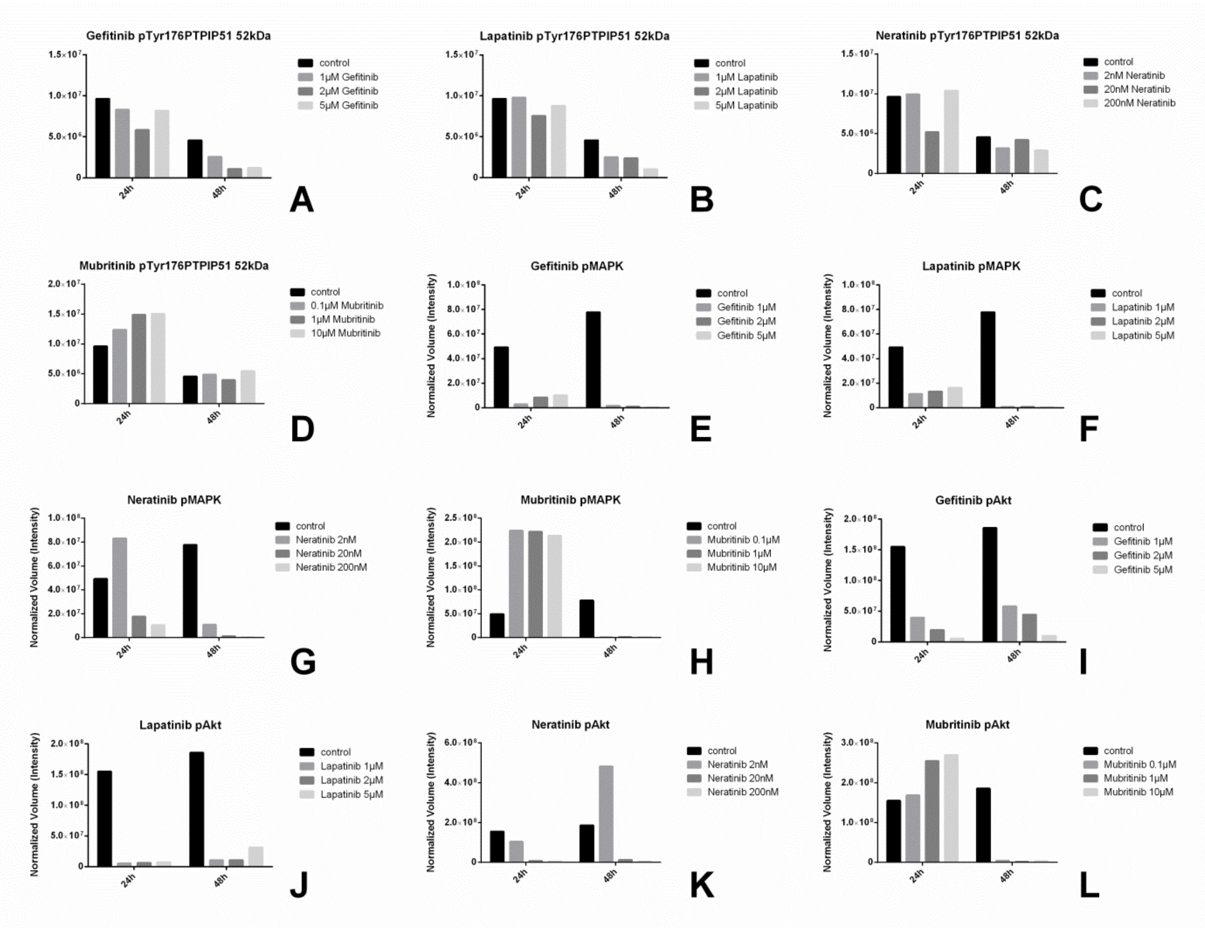

Supplementary Figure 7 **Evaluation of immunoblots of SK-BR3 cells under Gefitinib, Lapatinib, Neratinib and Mubritinib treatment for 24h and 48h.** Evaluation of pTyr176PTPIP51 (A-D). Evaluation of pMAPK (E-H). Evaluation of pAkt (I-L). Immunoblots were normalized to their corresponding stain free blot using ImageLab.

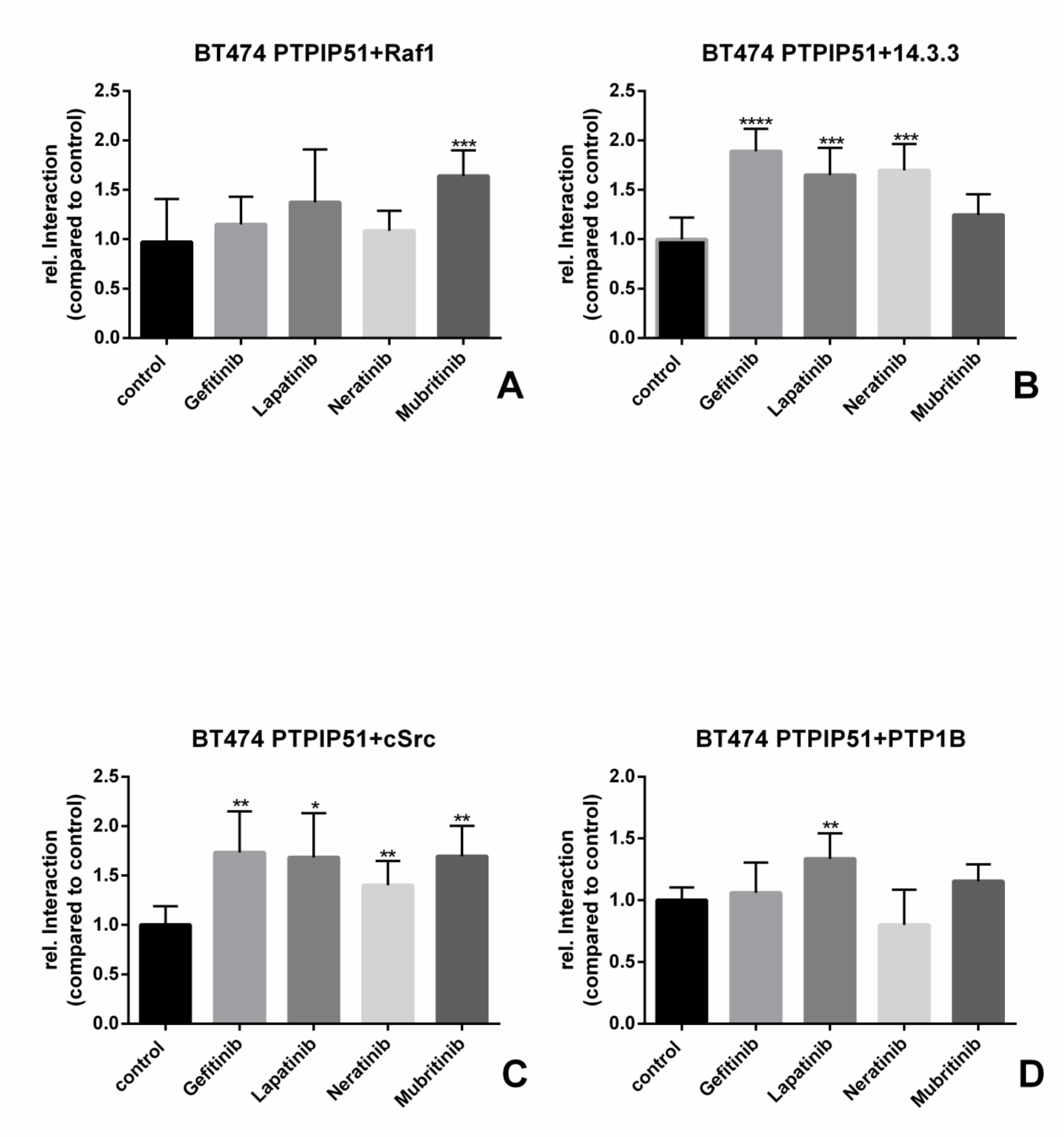

Supplementary Figure 8 **Interactions of PTPIP51 with different interaction partners in BT474 treated for 48h.** Interaction of PTPIP51 and Raf1 in BT474 treated with 5µM Gefitinib, 5µM Lapatinib, 200nM Neratinib or 10µM Mubritinib for 48h (A). Interaction of PTPIP51 and 14.3.3 in BT474 treated with 5µM Gefitinib, 5µM Lapatinib, 200nM Neratinib or 10µM Mubritinib for 48h (B). Interaction of PTPIP51 and cSrc in BT474 treated with 5µM Gefitinib, 5µM Lapatinib, 200nM Neratinib or 10µM Mubritinib for 48h (C). Interaction of PTPIP51 and PTP1B in BT474 treated with 5µM Gefitinib, 5µM Lapatinib, 200nM Neratinib or 10µM Mubritinib for 48h (D).
